# Activation of JNK and p38 MAPK Mediated by ZDHHC17 Drives Glioblastoma Multiforme Development and Malignant Progression

**DOI:** 10.7150/thno.40076

**Published:** 2020-01-01

**Authors:** Xueran Chen, Aijun Hao, Xian Li, Kaiqin Ye, Chenggang Zhao, Haoran Yang, Huihui Ma, Lei Hu, Zhiyang Zhao, Lizhu Hu, Fang Ye, Qiuyan Sun, Huaman Zhang, Hongzhi Wang, Xuebiao Yao, Zhiyou Fang

**Affiliations:** 1 Anhui Province Key Laboratory of Medical Physics and Technology; Center of Medical Physics and Technology, Hefei Institutes of Physical Science, Chinese Academy of Sciences, No. 350, Shushan Hu Road, Hefei, Anhui, 230031, China; 2 Department of Molecular Pathology, Hefei Cancer Hospital, Chinese Academy of Sciences, No. 350, Shushan Hu Road, Hefei, Anhui, 230031, China; 3 Key Laboratory of the Ministry of Education for Experimental Teratology, Department of Human Anatomy and Histoembryology, School of Basic Medical Sciences, Shandong University, No. 44, Wenhua Xi Road, Jinan, Shandong, 250012, China; 4 Department of Hand and Foot Surgery, Second Affiliated Hospital of Shandong University, No.247, Beiyuan Road, Jinan, Shandong, 250033, China; 5 University of Science and Technology of China, No. 96, Jin Zhai Road, Hefei, Anhui, 230026, China; 6 Department of Radiation Oncology, First Affiliated Hospital, Anhui Medical University, No. 218, Jixi Road, Hefei, Anhui, 230031, China; 7 Anhui Key Laboratory for Cellular Dynamics & Chemical Biology, University of Science and Technology of China, Hefei, Anhui, 230026, China

**Keywords:** glioblastoma multiforme (GBM), ZDHHC17, MAP2K4, JNK/p38, MAPK activation, tumorigenicity, stem cell self-renewal, genistein

## Abstract

**Rationale:** Glioblastoma multiforme (GBM) almost invariably gain invasive phenotype with limited therapeutic strategy and ill-defined mechanism. By studying the aberrant expression landscape of gliomas, we find significant up-regulation of *p*-MAPK level in GBM and a potent independent prognostic marker for overall survival. DHHC family was generally expressed in glioma and closely related to the activation of MAPK signaling pathway, but its role and clinical significance in GBM development and malignant progression are yet to be determined.

**Method:** Bioinformatics analysis, western blotting and immunohistochemistry (IHC) were performed to detect the expression of ZDHHC17 in GBM. The biological function of ZDHHC17 was demonstrated by a series of *in vitro* and *in vivo* experiments. Pharmacological treatment, flow cytometry, Transwell migration assay, Co- Immunoprecipitation and GST pulldown were carried out to demonstrate the potential mechanisms of ZDHHC17.

**Results:** ZDHHC17 is up-regulated and coordinated with MAPK activation in GBM. Mechanistically, ZDHHC17 interacts with MAP2K4 and p38/JNK to build a signaling module for MAPK activation and malignant progression. Notably, the ZDHHC17-MAP2K4-JNK/p38 signaling module contributes to GBM development and malignant progression by promoting GBM cell tumorigenicity and glioma stem cell (GSC) self-renewal. Moreover, we identify a small molecule, genistein, as a specific inhibitor to disrupt ZDHHC17-MAP2K4 complex formation for GBM cell proliferation and GSC self-renewal. Moreover, genistein, identified herein as a lead candidate for ZDHHC17-MAP2K4 inhibition, demonstrated potential therapeutic effect in patients with ZDHHC17-expressing GBM.

**Conclusions:** Our study identified disruption of a previously unrecognized signaling module as a target strategy for GBM treatment, and provided direct evidence of the efficacy of its inhibition in glioma using a specific inhibitor.

## Introduction

Glioblastoma multiforme (GBM) is the most prevailing and malignant type of primary brain tumor in adults and is associated with poor prognosis 1, 2. Approximately 88% of gliomas show changes in the mitogen activated protein kinase (MAPK) pathway, with MAPK genes determining the choice of invasive or proliferative phenotypes and, therefore, regulating metastases development and type 3, 4. An important fraction of glioblastoma (glioblastoma multiforme, GBM; Grade IV) exhibits high MAPK phosphorylation (*p*-MAPK). Unusual *p*-MAPK expression is a potent independent prognostic marker for poor overall survival (OS), and results in the increased tumor therapy resistance to radiation therapy or temozolomide (TMZ) 5, 6. The inhibitors targeting specific MAPKs, however, have not presented the therapeutic efficacy in glioma. Alternatively, multiple signaling pathways may converge at MAPKs or diverge from MAPK as a key downstream effector, which has triggered non-conventional strategies for targeting glioma cells such as methods aimed at signaling module alterations or the tumor microenvironment 7, 8.

ZDHHC17 (Zinc-finger DHHC-type containing 17) belongs to a super-family of palmitoyl-acyltransferases (PATs) catalyzing the palmitate attachment to other protein substrates. This post-translational modification plays an important role in regulating protein trafficking, subcellular localization, stability, interaction with effectors and other aspects of protein function 9, 10. ZDHHC17 is highly expressed in various embryonic cells, mainly nerve progenitor cells, but not in most adult tissues 11, 12. Although ZDHHC17 functioning directly as a PAT is best characterized, other functions have been implied based on non-palmitoylation-related studies. For example, ZDHHC17 potentially mediates Mg^2+^ transportation and may activate the c-Jun N-terminal kinase pathway 13, 14. Moreover, many current studies indicate pivotal roles of DHHC proteins and their substrates in tumorigenesis 12, 15, especially in malignant glioma development and progression. ZDHHC5 is up-regulated in p53-mutant glioma cells, and promotes their tumorigenicity and invasiveness 16. ZDHHC17 is situated in a novel glioma susceptibility locus on chromosome 11q12.1 17, 18. ZDHHC17 shows PAT action, particularly for the farnesylated-palmitoylation motif in proteins such as H-RAS, and its over-expression can allow cells to form rapidly developing tumors in mice 19. Nevertheless, a critical role of ZDHHC17 in glioma growth and progression is not known.

Here, we analyzed ZDHHC17 function in recruiting MAP2K4 and JNK/p38 to build a signaling module for MAPK pathway activation, the relevance of this interaction for malignant GBM progression, and identified an inhibitor targeting the ZDHHC17-MAP2K4 module.

## Methods

### Plasmids, Cell Culture, and Stable Cell Line Generation

U251, U87MG, CCF-STTG1, U118MG, SW1783, SW1088, A172, and SH-SY5Y glioma cell lines were obtained from the American Type Culture Collection (ATCC) during 2012 to 2014, and were characterized with DNA fingerprinting and isozyme detection. All the lines were revived every three to four months, being maintained at low passages for experimental utilization. Normal human astrocytes (NHAs) were obtained from Lonza (Walkersville, MD USA) in 2017. All the cell lines used in the study were authenticated regularly with the usage of morphological observation and were inspected as being without mycoplasma contamination. In March 2018, they were inspected for mycoplasma for the last time.

Glioma cell lines were cultured in DMEM supplemented with 1% (100×) streptomycin/ penicillin and 10% FBS. NHAs were cultured similarly to human glioblastoma multiforme cell lines. GSCs were cultured in DMEM/F12 including 2.5 mg/mL heparin, 2% B27 supplement (% vol), 20 ng/mL EGF, and 20 ng/mL bFGF. The culture medium was replaced every 10 days and the reagents of EGF and bFGF were added twice a week. Since the first establishment, the experimentations with neurospheres were conducted with lines cultured for fewer than 20 passages. According to Chen et al. 16, as delineated previously, an *in-vitro* cell viability assay and *in-vitro* limiting dilution neurosphere formation assay were carried out. hNSCs were cultured as for GSCs. The cells were cultured without growth factors or with 10% FBS to induce the differentiation of hNSCs and GSCs.

Full-length *ZDHHC17* or *MAP2K4* cDNA was cloned in a pcDNA3.0 expression vector (Invitrogen) for overexpression studies. Subsequently, based on manufacturer instruction, the vector construct was transfected into cells using Lipofectamine™ 3000 transfection reagent. The cells were transfected with Negative, MAP2K4 (#SR304323, Origene) or ZDHHC17 (#SR323571, Origene) Stealth siRNA per manufacturer instruction using Lipofectamine® RNAiMAX reagent for knockdown experimentation. In addition, cells were transduced with pGFP-C-shLenti virus specks particularly for control shRNA or ZDHHC17 (#TG300348, Origene). The cells were treated with puromycin (0.5 μg/mL; #A1113802, Thermo Fisher Scientific) for establishment of stable cell lines over one week.

### Glioma Tissue Microarray and Immunohistochemistry (IHC) Staining

Glioma tissue microarrays were obtained from U.S. Biomax, Inc. The IHC analyses of glioma tissue microarrays were conducted as delineated previously 20. Briefly, staining results were visualized by the KF-PRO Digital Slide Scanning System (Kongfong Biotech International Co., LTD; Ningbo, China). Negative control was treated in the same way without adding the primary antibodies. Results of immunohistochemistry staining were evaluated by two independent pathologists with patient characteristics' no prior knowledge. Discrepancies were dissolved by consensus. The staining extent score was on a scale of 0-4, corresponding to immunoreactive tumor cells' percentage (0%, 1%-5%, 6%-25%, 26%-75%, and 76%- 100%, respectively). The staining intensity was scored as negative (score=0), weak (score=1), or strong (score=2). A score that ranged from 0-8 was calculated by multiplying the staining extent score with the intensity score, leading to a low (0-4) level or a high (6-8) level value for each specimen. ZDHHC17^high^/ MAP2K4^high^ were defined as both the score of ZDHHC17 and MAP2K4 are the high (6-8) level in the same specimen.

### Real-time Quantitative PCR (RT-qPCR)

Using the RNeasy kit (Qiagen), total RNA was prepared, then transcribed to cDNA with the iScript Reverse Transcription Supermix and amplified with Taq PCR Master Mix. Utilizing β-actin as the internal control, every sample was prepared in triplicate. Reverse and forward primer sequences were follows: *ZDHHC17*, 5'-TGGATCAACTTGGAGGGGAC-3' and 5'-TTTTGCTTGCCTTGCCTCTT-3'; *MAP2K4*, 5'-TCTGTGACTTCGGCATCAGT-3' and 5'-TGGGAGTAGCTGGCATTTGA-3'; and β-actin, 5'- CTATCCCTGTACGCCTCTGG-3' and 5'-CGTACAGGTCTTTGCGGATG-3'.

### Western Blot (WB) and Immunoprecipitation (IP)

WB and IP were performed as discussed previously 21. Briefly, cells were collected and lysed in RIPA buffer encompassing phosphatase inhibitor cocktail and 1% protease on ice for 0.5 h and cleared by centrifugation at 12,000 rpm and 4°C for 900 s. Using 1 μg of agarose-immobilized antibody, the whole protein lysate (500 μg) was incubated for immunoprecipitation at 4 °C overnight. The proteins subjected to co-IP and IP with the usage of sodium dodecyl sulfate-polyacrylamide gel electrophoresis (SDS-PAGE) were resolved and then transferred to a polyvinylidene difluoride membrane probed with primary antibodies at 4 °C for 16 h. Subsequently, they were blocked with 5% skim-milk/0.1% Tween-20 in Tris-buffered saline at 37 °C for 60 min. After detection using improved chemiluminescence, a horseradish peroxidase-conjugated secondary antibody was utilized (Pierce).

### Glutathione S-transferase (GST) Pull-down Assay

To obtain GST-MAP2K4 as GST fusion protein for the GST pull-down assay, *MAP2K4* cDNA was cloned in-frame into the pGEX6p-1 vector. Following immobilization on glutathione-sepharose beads (#G0924, Sigma-Aldrich), the fusion proteins of GST-MAP2K4 and GST were incubated with ZDHHC17 FLAG-expressing HEK293 cell lysates. After washout, the bound proteins were resolved by SDS-PAGE and WB.

### Immunofluorescence Analysis

U118MG cells were fixed with paraformaldehyde (4%), washed with phosphate-buffered saline (PBS), and incubated in blocking buffer (1×PBS including 0.3% Triton X-100 and 5% normal goat serum) for 60 min. Subsequently, the samples were incubated with primary antibodies at 4 °C overnight followed by detecting Alexa 568 goat anti-rabbit (Invitrogen) and Alexa 488 goat anti-mouse (Invitrogen) secondary antibodies. Nuclei were counterstained with 4',6-diamidino-2-phenylindole (Biotechnology's Beyotime Institute), and the samples were mounted with coverslips fixed using fluorescence mounting medium (Biotechnology's Beyotime Institute). The images were obtained using a fluorescence microscope (IX71; Olympus) and then adapted for contrast and brightness with the usage of the Image-Pro Plus 6.0 software (Media Cybernetics).

### Colony Formation Assay

According to Yamashita et al. 22, colony formation assays were performed. The cells were plated at 500 cells/well in a 10-cm plate, then grown for 10 days in standard growth medium and washed with PBS. The cells were fixed in cold methanol for 20 min, washed, and stored. Fixed cell colonies were visualized by incubating the cells with 0.5% (w/v) crystal violet for 0.5 h. Extra crystal violet was removed by washing with PBS. The visible colonies, consisting of ≥50 cells, were counted.

### Cell Cycle Analysis

Following Cytomics FC500 Flow Cytometer CXP analysis, cell cycle status was assayed via propidium iodide (PI) staining. The cell cycle profiles were determined using software of CXP analysis (Beckman Coulter Inc.).

### Invasion and Transwell Migration Assay

Transwell chambers with 8-μm pores (Corning) were utilized to assess cell migration. The Transwell membrane was previously coated with Matrigel matrix (30 μL) for the invasion assay of tumor cells (1:3 mixed with PBS; BD Biosciences). Cells (1 × 10^5^) were loaded to the top chamber of the Transwell dish (8 μm pore size; Corning Inc.). FBS (10%) was loaded in the bottom chamber as a chemoattractant. After 16h, the cells in the bottom chamber were fixed and then stained with 0.005% (w/v) Crystal Violet. The number of invaded or migrated cells was measured by counting those five random areas in every membrane.

### *In Vitro* Kinase Assay with Recombinant MAP2K4 and ZDHHC17

We used the *ex vivo* kinase assay system from Millipore Upstate Biotechnology according to manufacturer instruction with some modification. The chemical compounds as noted below were added into the kinase buffer at 4 °C to a final volume of 20 μL: ZDHHC17 (50 ng); recombinant MAP2K4 (0.05 U); a no genistein control or genistein at 80, 40, 20, 10, 5.0, 1.0, 0.1, or 0.01 μM; JNK2 (1 μg); ATP (2.5 μM); or MgCl_2_ (375 μM). All reactions were incubated together. Diminishing and denaturing gel loading buffer with a final concentration of SDS (1%), Tris-HCl (125 mM) at pH 6.8, dithiolthreitol (0.8%), bromophenol blue (0.05%), and 5% (v/v) glycerol was added and incubated for 5 min at 95 °C, then for 5 min at 4 °C, and results were determined using WB after incubation for 5 min at 30 °C.

### Xenograft Tumor Model

Six-week-old female C57BL/6 mice, about 18-25 g, were anesthetized by intraperitoneal administration of ketamine (132 mg/kg) and xylazine (8.8 mg/kg) and subcutaneously injected at the area of upper left flank with the cell suspension (0.1 mL) consisting of 5 × 10^3^ GSCs originated from U118MG. These mice were injected with 100 mg/kg genistein every two days by means of the tail vein after five days or X-irradiated with 20 Gy (4.5-4.6 Gy/min) for tumor, administered by gastric infusion with TMZ (50 mg kg^-1^ day^-1^). At the inoculation site, the width and length of the tumor mass were measured to assess the tumor development.

GSCs stemming from the cells of U118MG were transduced with lentivirus containing luciferase prior to intracranial implantation. Subsequently, stereotactic injection was utilized to inject the suspension of 2 × 10^5^-cells/mL cell (2 μL) in PBS with high-glucose to the hemi-striatum of BALB/c mice at six-weeks of age. The co-ordinate parameters as below were adopted: dorso-ventral = -3.5 mm; medio-lateral = +2.5 mm; antero-posterio r= 0. Luciferin was injected to the peritoneal cavity to track the tumor cells* in vivo* in the post-injection period of around 6 weeks. Animals were anesthetized with sodium pentobarbital (50 mg/kg) and the IVIS Lumina system was utilized for bioluminescence imaging (Perkin-Elmer).

### Molecular Docking for Protein Binding

To study the binding mode between the human MAP2K4 and the human ZDHHC17 using the server ZDOCK, molecular docking was conducted. The MAP2K4 (PDB ID: 3VUT) and the three-dimensional (3D) structures of human ZDHHC17 (PDB ID: 3EU9) were downloaded from the Protein Data Bank. The X-ray structure of the ZDHHC17 ANK domain could be downloaded from RCSB Protein Data Bank (PDB code: 3EU9, resolution: 1.99 Å). UCSF Chimera was utilized to construct the 3D structure of genistein as follows, via the minimization of energy. The parameters of the default option were utilized as described in the ZDOCK server for docking. Based on the docking score, the best rated structure was subject to visual analysis using the software PyMoL 1.7.6.

### Statistical Analysis

All the statistics are shown as the means ± SD. The number of replicates for every experiment was expressed in the figure legends. A 2-tailed *t* test was utilized to evaluate the statistical differences between the two groups. The comparison of multiple groups was conducted with the usage of a 1-way ANOVA followed by Dunnett's post-hoc test. The extreme limiting dilution analysis (ELDA) was applied to study the* in vitro* clonogenicity assays. A log-rank analysis was conducted to determine the importance of the Kaplan-Meier survival plot. *R* software, SPSS 19.0 software and GraphPad Prism 6 were utilized to conduct the statistical analysis. A *p* value under 0.05 was regarded as statistically significant.

## Results

### The ZDHHC17-MAP2K4-JNK/p38 Signaling Module Regulates JNK/p38 Activation in GBM

**Figure 1:**
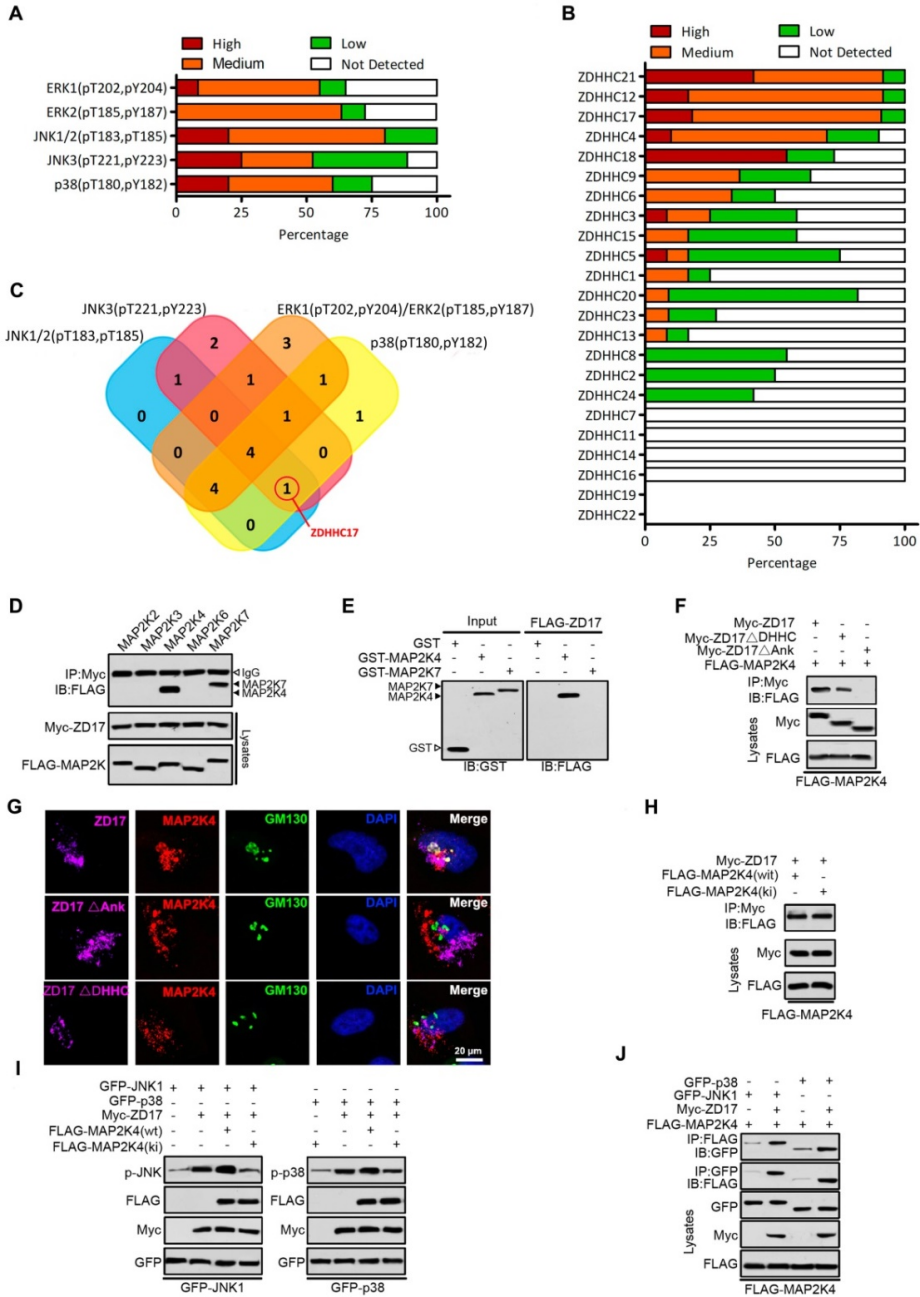
** ZDHHC17-MAP2K4-JNK/p38 Signaling Module Formation in Glioblastoma Multiforme (GBM). (A)** The expression of ERK1 (pT202, pY204), ERK2 (pT185, pY187), JNK1/2(pT183, pT185), JNK3 (pT221, pY223) and p38 (pT180, pY182) in GBM is summarized based on the immunohistochemistry results of the Human Protein Atlas. **(B)** The expression of different DHHCs in glioma is summarized based on the immunohistochemistry results of the Human Protein Atlas. **(C)** Venn diagram showing the relationship between expression patterns of different DHHCs and activation of ERK1, ERK2, JNK1/2, JNK3, and p38 in GBM. **(D)** Lysates from HEK293 cells expressing Myc-ZDHHC17 and Flag-MAPKKs were subjected to immunoprecipitation (IP), followed up by immunoblotting (IB) with anti-FLAG antibodies and anti-Myc. IP, immunoprecipitation; IB, immunoblotting. **(E)** GST pull-down utilizing purified GST-MAP2K4 (or GST-MAP2K7) and FLAG-ZDHHC17-expressing HEK293 cell lysates, followed by IB with an anti-FLAG antibody. **(F)** ZDHHC17 associates with MAP2K4 via ZDHHC17 ANK rather than PAT activity domain. IP of lysates from HEK293 cells expressing Flag-MAP2K4 and Myc-ZDHHC17 mutants, followed by IB with anti-Flag antibodies and anti-Myc. ZDHHC17 ΔDHHC: ZDHHC17 aa 440-487 deletion; ΔANK: ZDHHC17 aa 51-288 deletion. **(G)** ZDHHC17 protein recruits MAP2K4 in the Golgi and cytoplasmic vesicles in U118MG cells, in a ZDHHC17 ANK domain-dependent manner. Immunofluorescence of U118MG cells expressing Myc-ZDHHC17 (or -ZDHHC17 ΔDHHC or ΔANK) with anti-Myc (Pink), MAP2K4 (Red), and GM130 (Green) antibodies. Scale bar, 20 µm. **(H)** ZDHHC17 interacts with MAP2K4 wild-type (wt) and the kinase-inactive mutant (ki). IP of lysates from HEK293 cells expressing Myc-ZDHHC17 and FLAG-MAP2K4wt (or FLAG-MAP2K4ki), followed by IB with anti-FLAG antibodies and anti-Myc. **(I)** MAP2K4 contributes to the ZDHHC7-mediated JNK1 and p38 phosphorylation. FLAG-MAP2K4ki co-expression in HEK293 cells reduces Myc-ZDHHC17-mediated GFP-JNK1 (or GFP-p38) phosphorylation. **(J)** MAP2K4 and JNK1 (or p38) are recruited by ZDHHC17 in a signaling module. GFP-JNK1 (p38) and FLAG-MAP2K4 were introduced in HEK293 cells, with or without Myc-ZDHHC17. MAP2K4 presence upon JNK1 (or p38) IP is improved by ZDHHC17 co-expression.

As reported, the MAPK pathway has been activated in over 88% of gliomas, and various cellular functions are regulated by MAPK signaling module, including proliferation, differentiation and malignant transformation in gliomas 3, 4. Firstly, the expression ERK1(pT202, pY204), ERK2(pT185, pY187), JNK1/2(pT183, pT185), JNK3(pT221, pY223) and p38(pT180, pY182) in GBM was evaluated based on the Human Protein Atlas (**Figure 1**A). ERK1, ERK2, JNK1/2, JNK3 and p38 were unusually activated (> 50%) in GBM, respectively. DHHC family was generally expressed in glioma (**Figure 1**B), and closely related to the activation of MAPK signaling pathway **(Figure S1**-**S5**). Only ZDHHC17, as a candidate gene for experimental validation, might related to both JNK and p38 activation (**Figure 1**C).

As ZDHHC17 promotes JNK/p38 phosphorylation and activity, the function of several mitogen- activated protein kinase kinases (MAPKKs), upstream activators of JNK/p38, was examined in a predicted signaling module (**Figure 1**D). Upon MAPKK expression in HEK293 cells, ZDHHC17 interacts with MAP2K4 (JNK/p38 activator) and MAP2K7 (JNK activator), but not MAP2K2 (ERK activator) or MAP2K6 and MAP2K3 (p38 activators). Indeed, ZDHHC17 strongly bound to GST-MAP2K4* in vitro*, but MAP2K7 functional involvement was not supported (**Figure 1**E). The association with MAP2K4 was independent of ZDHHC17 PAT activity, because both ZDHHC17 wild-type (wt) and ΔDHHC could bind MAP2K4 by IP (**Figure 1**F). The ZDHHC17 and MAP2K4 interaction mostly depended on the ZDHHC17 ankyrin-repeat (ANK) domain, as ZDHHC17 ΔANK lost the ability of binding MAP2K4 (**Figure 1**F). Consistent with immunofluorescent analysis, ZDHHC17 ΔDHHC still co-localized with MAP2K4 at the Golgi apparatus, similar to ZDHHC17, but not ZDHHC17 ΔANK (**Figure 1**G). The kinase-dead mutant MAP2K4ki still interacts with ZDHHC17, but leads to attenuated JNK/p38 phosphorylation (**Figures 1**H and **1**I). Furthermore, JNK or p38 association with MAP2K4 was markedly increased in the presence of ZDHHC17 (**Figure 1**J). These results suggest that ZDHHC17 recruits MAP2K4 and JNK/p38 to build a signaling module for JNK/p38 activation.

### The ANK Domain of ZDHHC17 Is Responsible for Interaction with MAP2K4

**Figure 2:**
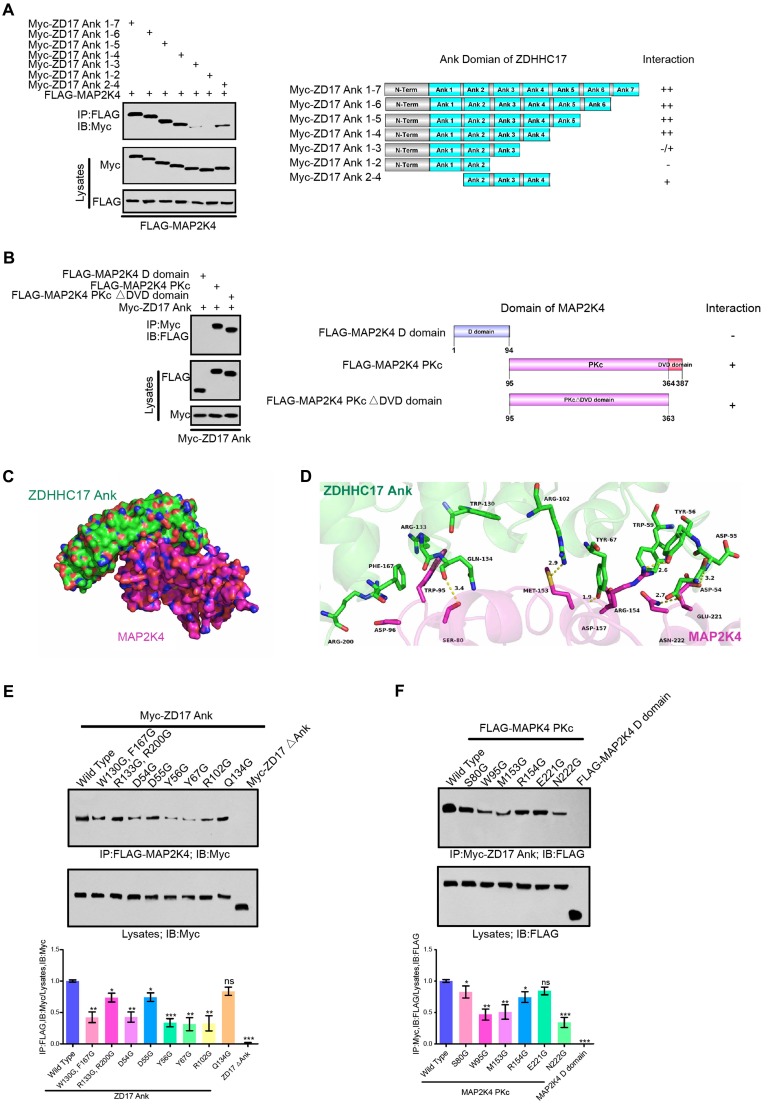
** ZDHHC17 and MAP2K4 Interact Via Specific Binding Motifs. (A)** ZDHHC17 ANK (2-4) domain is crucial for ZDHHC17-MAP2K4 interaction. Myc-tagged ZDHHC17 ANK (1-7) and various deletions (right). Interaction capability (positive or negative) is shown.** (B)** MAP2K4 PKc domain is crucial for the ZDHHC17-MAP2K4 interaction, independent of the DVD domain. FLAG-tagged MAP2K4 and various MAP2K4 deletion fragments (right). Interaction capability (positive or negative) is shown.** (C)** Surface representation of the complex. ZDHHC17 and MAP2K4 binding mode molecular docking was performed using the ZDOCK server. MAP2K4 (Magenta) binds to the concave ANK(1-7) (Green) region between ANK2 and ANK4.** (D)** Cartoon and stick representation of ZDHHC17 ANK (1-7) (Pale Green) and ANK (1-7) (Green), respectively.** (E)** IP of lysates from HEK293 cells expressing Flag-MAP2K4 and Myc-ZDHHC17 ANK (1-7) mutants, followed by IB with anti-Flag antibodies and anti-Myc. Data represent the means ± SD from three separate experiments (*ns*, not significant; *p < 0.05; **p <0.01; ***p < 0.001, unpaired* t-*test).** (F)** IP of lysates from HEK293 cells expressing FLAG-MAP2K4 mutants and Myc-ZDHHC17, followed by IB with anti-Flag antibodies and anti-Myc. Data represent the means ± SD from three separate experiments (*ns*, not significant; *p < 0.05; **p < 0.01; ***p < 0.001, unpaired *t*-test).

The ANK domain of ZDHHC17 is the key region interacting with MAP2K4. To map the binding motifs of ZDHHC17 with MAP2K4, the deletion mutants of ZDHHC17 in ANK domain were produced and the affinity to MAP2K4 were validated (**Figure 2**A). Deleting the motif of ANK(5-7) did not affect the ZDHHC17-MAP2K4 interaction but removing ANK(4) diminished it. Strikingly, the deletion of ANK(3-4) from ZDHHC17 ANK domain completely disrupted the interaction. ANK(1) deletion from ZDHHC17 ANK(1-4) slightly reduced the interaction with MAP2K4. Thus, these results suggested that MAP2K4 binds specifically to the ANK(1-4) domain with a preference for ANK(2-4) in ZDHHC17. And ZDHHC17 also binds specifically to the MAP2K4 protein kinase c (PKc) domain, relative to its N-terminal docking (D) domain (**Figure 2**B).

Molecular docking was carried out to dissect the binding mode between the human ZDHHC17 and MAP2K4 by utilizing the ZDOCK server. The three-dimensional (3D) human ZDHHC17 (PDB ID: 3EU9) and MAP2K4 (PDB ID: 3VUT) structures were downloaded from Protein Data Bank. It was shown that the interaction between ZDHHC17 (green) and MAP2K4 (magenta) (**Figure 2**C). A hydrophobic interaction was observed between the residues of Trp-130 and Phe-167 in ZDHHC17 with the residue of Trp-95 at MAP2K4 (**Figure 2**D). More detailed analysis showed that ZDHHC17 residues Arg-133 and Arg-200 formed cation-π interactions with MAP2K4 residue Trp-95. In addition, a π-π stacking interaction was observed between ZDHHC17 residue Phe-167 and MAP2K4 residue Trp-95. Notably, six hydrogen bond interactions were shown between ZDHHC17 residue Gln-134 and MAP2K4 Ser-80 (bond length: 3.4 Å), ZDHHC17 Tyr-67 and Arg-102 and MAP2K4 Met-153 (bond length: 1.9 and 2.9 Å), ZDHHC17 Tyr-56 and MAP2K4 Arg-154 (bond length: 2.6 Å), ZDHHC17 Asp-54 and MAP2K4 Asn-222 (bond length: 2.7 Å), and ZDHHC17 Asp-55 and MAP2K4 Glu-221 (bond length: 3.2 Å), comprising the main binding affinity between ZDHHC17 and MAP2K4. These molecular simulations rationally explain the interface involved in ZDHHC17-MAP2K4 interaction. Thus, we generated a series of mutants in ZDHHC17 or MAP2K4 and examined the ability to disturbing the affinity between ZDHHC17 or MAP2K4 (**Figures 2**E and **2**F). The results showed that the ZDHHC17- MAP2K4 interaction could mostly be attributed to ZDHHC17 residues Trp-130, Phe-167, Asp-54, Tyr-56, and Tyr-67 and MAP2K4 residues Trp-95, Met-153, and Asn-222.

### ZDHHC17 and MAP2K4 Are Highly co-expressed in GBM and Associate with Poor Prognosis in Patients with GBM

**Figure 3:**
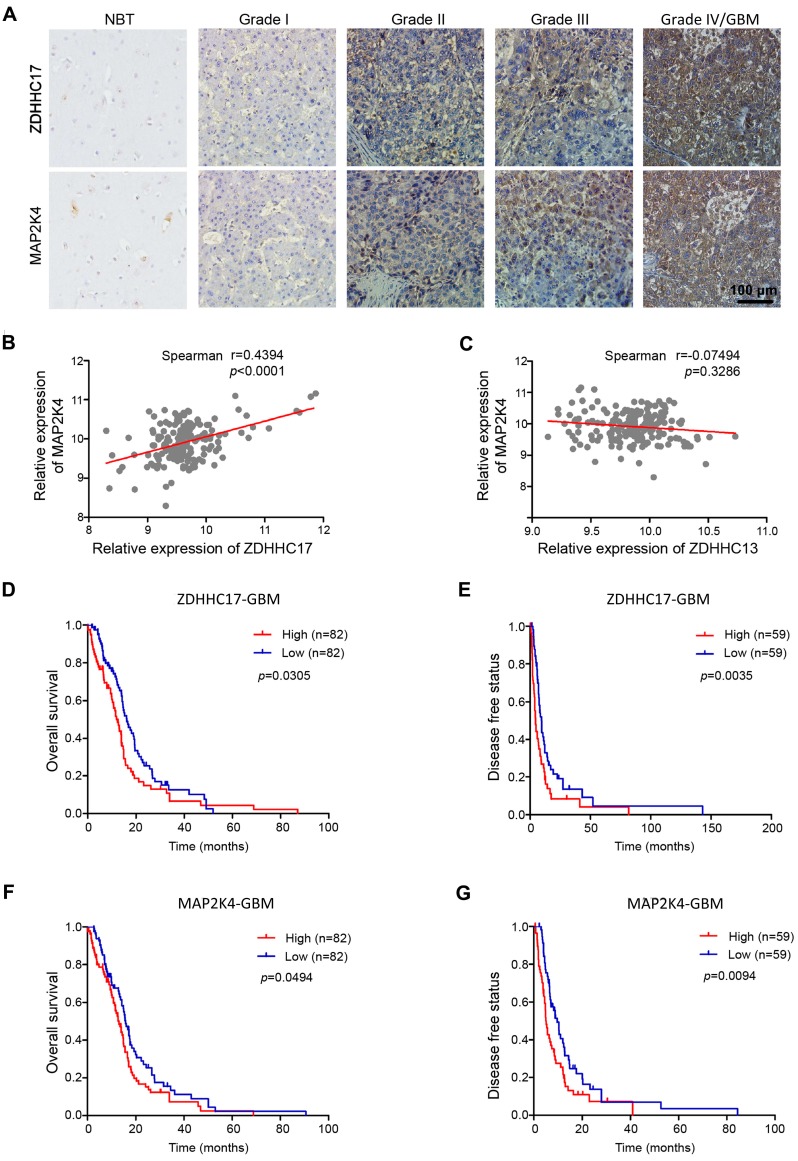
** ZDHHC17 and MAP2K4 Expression Correlates with Glioma Tumor Grade and has Prognostic Significance**. **(A)** Representative images of immunohistochemistry staining for ZDHHC17 and MAP2K4 in different grade gliomas and normal brain specimens. Scale bar, 100 µm. **(B, C)** Correlation between *MAP2K4* and *ZDHHC17* (B) or *ZDHHC13* (C) in GBM using TCGA datasets. Correlation statistical significance was evaluated using a linear regression model (n = 171, *r* = 0.4394, *p* < 0.001) (B), (n = 172, *r* = -0.07494, *p* = 0.3286) (C). **(D-G)** Accumulative overall (D; F, n=164) and disease-free survival (E; G, n=118) of patients with high or low ZDHHC17 (D, E) or MAP2K4 (F, G) expression GBM (based on IHC staining results) calculated using the Kaplan-Meier method and compared with the log-rank test for the same set of patients (*p < 0.05; **p < 0.01).

Next, we checked ZDHHC17 and MAP2K4 expression in a glioma tissue microarray (TMA) by immunohistochemistry. Expression of both ZDHHC17 and MAP2K4 was progressively elevated in glioma samples from grade I to grade IV *versus* normal brain tissues, and was highly correlated (**Figures 3**A and **3**B). Attesting to its specificity, the expression of ZDHHC13, the homeodomain protein of ZDHHC17, was not related to MAP2K4 expression in glioma tissue (**Figure 3**C). Kaplan-Meier survival analysis showed that patients with highly ZDHHC17- expressing glioma had shorter disease-free survival (DFS) and OS than the low-expression groups; similar results were also obtained for patients with highly expression of MAP2K4 (**Figures 3**D-**3**G). Consistent with these results, patients with highly co-expression of ZDHHC17 and MAP2K4 were significantly inversely correlated with both DFS and OS (**Figure S6**).

### ZDHHC17 Is Associated with Glioma Occurrence

**Figure 4:**
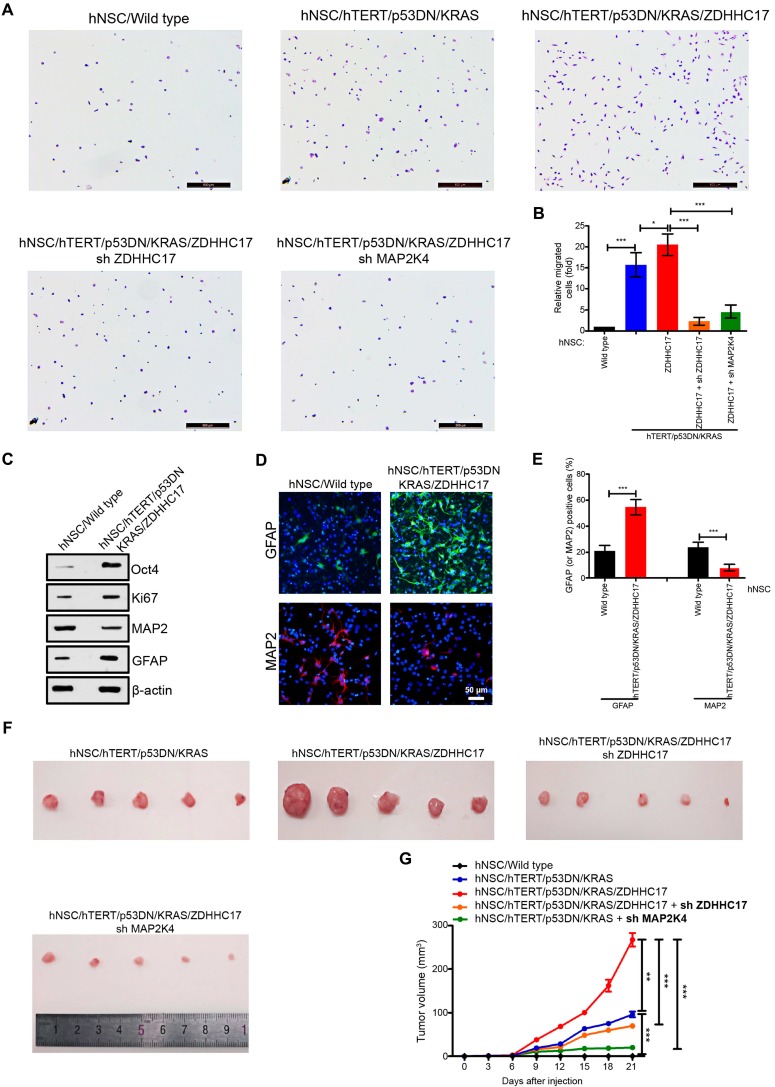
** ZDHHC17 is Important for Brain Glioma Development. (A, B)** Migratory human neural stem cells (hNSCs) transfected with indicated lentiviruses in Transwell assays. Cells were stained with crystal violet for counting. Scale bar, 500 µm. Data represent the means ± SD from three separate experiments (*p < 0.05; ***p < 0.001, unpaired* t*-test).** (C)** Western blot of markers Oct 4 (stem cell self-renewal), Ki67 (proliferative), and GFAP (glial) and MAP2 (neuronal) in TERT/CA-KRAS/DN-p53/ZDHHC17 hNSCs compared to hNSCs.** (D, E)** Immunofluorescence (D) and positive percentage (E) of GFAP and MAP2 in TERT/CA-KRAS/DN-p53/ZDHHC17 compared to hNSCs. Scale bar, 50 μm. Data represent the means ± SD from three separate experiments (**p < 0.01; ***p < 0.001, unpaired* t-*test).** (F, G)** C57BL/6 mice were subcutaneously injected with 1 × 10^5^ hNSCs transfected with indicated lentiviruses. Tumor volumes were measured every three days. Each point represents the mean volume ± SD of five tumors (**p < 0.01; ***p < 0.001). After 3 weeks, nude mice were sacrificed; the dissected tumors are shown.

Whether aberrant ZDHHC17 expression is linked to glioma incidence, human neuronal stem cells (hNSCs) were co-transfected with the plasmids encoding TERT, constitutively activated (CA)-KRAS, dominant-negative (DN)-p53, and ZDHHC17. TERT, KRAS, and p53 mutation or their abnormal expression are common in malignant glioma, and can endow hNSCs with neoplastic features 16, 23, 24. Gene Set Enrichment Analysis (GSEA) revealed that hNSCs harboring TERT/CA-KRAS/DN-p53/ZDHHC17 showed a more obvious glioma signature than control hNSCs (**Figure S7**). These hNSCs were also significantly invasive than control or TERT/CA-KRAS/DN-p53 hNSCs (**Figures 4**A and **4**B), whereas it was markedly reduced upon the decreased expression of ZDHHC17 or MAP2K4 with shRNA knockdown. Furthermore, TERT/CA-KRAS/DN-p53/ZDHHC17 hNSCs exhibited higher proliferative capacity and stem cell characteristics, although their differentiation potential was altered as evidenced GFAP up-regulation relative to the control hNSCs (**Figures 4**C-**4**E).

The role of ZDHHC17 in glioma growth was also evaluated using a tumor formation assay (**Figures 4**F and **4**G). Tumors were produced by hNSCs with co-expressing TERT/CA-KRAS/DN-p53, but not CA-KRAS or TERT alone. Nevertheless, tumor formation ability was higher for TERT/CA-KRAS/DN-p53/ZDHHC17-expressing hNSCs, which yielded tumors with a 3-fold higher volume. Conversely, MAP2K4 or ZDHHC17 knockdown TERT/CA-KRAS/DN-p53/ZDHHC17 hNSCs often resulted in smaller tumors, suggesting that ZDHHC17 contributes to glioma development.

### Screening of Genistein as a ZDHHC17-MAP2K4 Signaling Module Inhibitor

**Figure 5:**
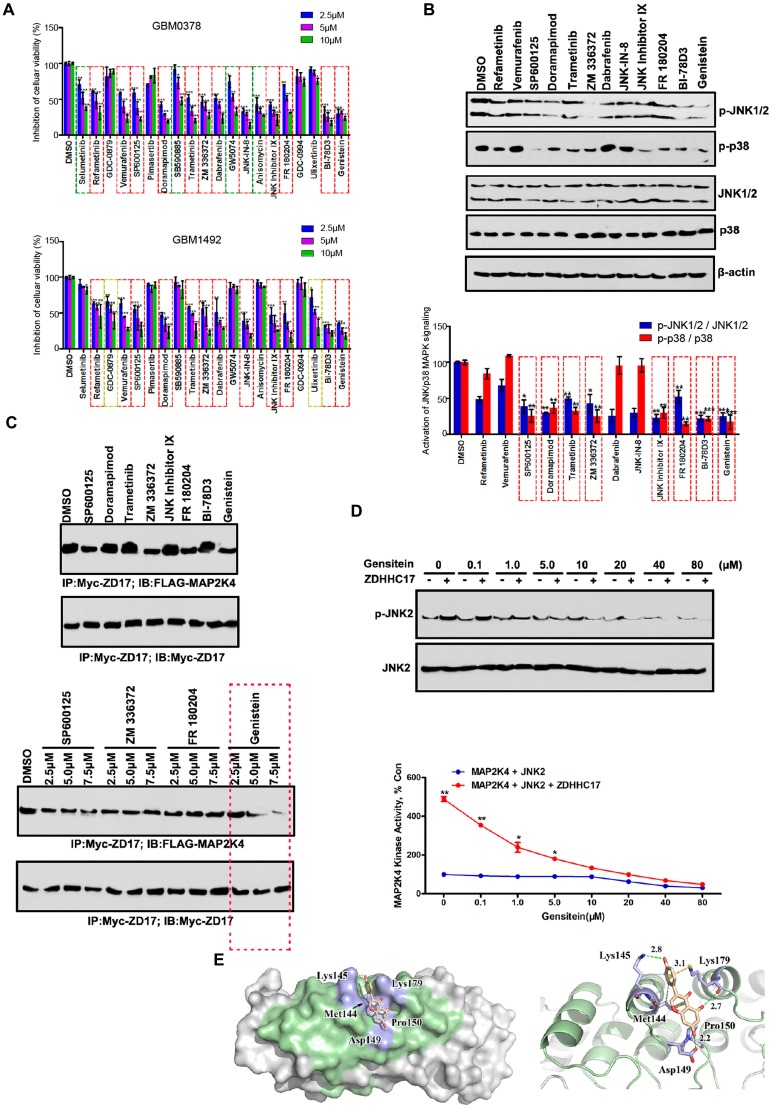
** Genistein is a Lead Candidate for Inhibiting the ZDHHC17-MAP2K4 Interaction in GBM. (A)** GBM0378 and GBM1492, from patients with GBM, cell viability after 24h-treatment with indicated inhibitors at indicated concentration from the MAPK Compound Library. Data represent the means ± SD from three separate experiments (**p < 0.01; ***p < 0.001, unpaired *t*-test).** (B, C)** Western blot and quantitation of JNK1/2 and p38 phosphorylation; β-actin was used as a loading control (B), and immunoprecipitation (IP) of the ZDHHC17-MAP2K4 interaction (C) after 24h-treatment with indicated inhibitors (5.0 μM for each inhibitor) in ZDHHC17-expressing U118MG cells. Data represent the means ± SD from three separate experiments (*p < 0.05; **p < 0.01; ***p < 0.001, unpaired *t*-test).** (D)** Genistein treatment directly inhibits ZDHHC17-MAP2K4 kinase activity. ZDHHC17-MAP2K4 kinase activity in the presence of genistein (0-80 μM) measured with commercially available kits using a passive JNK2 as the substrate. The phosphorylated substrate was tested by western blot and band density calculated using AlphaEaseFc software. Data represent the percentage of activity in the untreated control reaction; the means ± SD from three separate experiments are shown (*p < 0.05; **p < 0.01, unpaired *t*-test).** (E)** Surface representation of the complex. Molecular docking of the binding mode between ZDHHC17 (Green) and genistein was performed using the ZDOCK server.

ZDHHC17 mRNA and protein levels were increased in GBM cell lines compared to those in normal human astrocytes (NHAs, **Figures S8**A, and **S8**B). We sought to screen clinically applicable small molecules that selectively inhibited ZDHHC17-MAP2K4 activity in GBM. The drug library was composed of 65 small molecule inhibitors included in the clinical guideline or current clinical trials for the treatment of abnormal MAPK-associated signaling pathway activity. As shown in **Figure S8**C, the proliferation of SW1088 cells (ZDHHC17 low-expression cell line) was affected by 65 MAPK inhibitors, to different degrees, whereas only 20 significantly inhibited U118MG (ZDHHC17 high-expression cell line) cell proliferation. To characterize inhibitor efficacy, we also investigated 20 inhibitors using different ZDHHC17-positive patient-derived glioblastoma sphere samples (**Figure 5**A), finding that 12 exhibited therapeutic effects. Accordingly, only 8 inhibitors significantly reduced both JNK and p38 phosphorylation in U118MG cells (**Figure 5**B). Among these, genistein specifically inhibited the ZDHHC17 and MAP2K4 interaction (**Figure 5**C), and was therefore selected as the lead candidate for ZDHHC17-MAP2K4 inhibition and GBM therapy.

Next, we investigated whether genistein directly suppresses ZDHHC17-activated MAP2K4 kinase activity using MAP2K4 and purified ZDHHC17 in an *ex vivo* kinase assay with a recombinant JNK2 as the substrate. With increasing genistein concentration, JNK2 phosphorylation level decreased. Genistein specifically suppressed ZDHHC17-MAP2K4 kinase activity with an average half maximum inhibitory concentration (IC_50_) of 0.80 μM (**Figure 5**D).

To investigate whether genistein suppressed MAP2K4 kinase activity via directly blocking the interaction with ZDHHC17, the computer-assisted modeling was used to estimate the mechanism. We calculated all conceivable positions in three-dimensional space, or conformations, by which genistein could interact with the ZDHHC17 ANK(2-4), as evaluated by the genetic algorithm applied in the AutoDock program, and selected the highest scoring conformation (**Figure 5**E). In this predicted model for genistein-ZDHHC17 binding, the cleft was spanned by genistein between the domains by making hydrogen bonds with Lys-145, Asp-149, and Lys-179. Detailed analysis also showed that the phenolic benzene ring of genistein formed cation-π interactions with ZDHHC17 residue Lys-179 as well as a hydrophobic interaction between ZDHHC17 residues Lys-145 and Met-144. Thus, we generated a series of mutants in ZDHHC17 and examined the ability of genistein to disturbing the affinity between ZDHHC17 and MAP2K4. Mutants of these residues would have a strong impact on the genstein's disturbing the affinity between ZDHHC17 and MAP2K4 (**Figures S9**A), and could gain resistance to genstein (**Figures S9**B). The results showed that the genistein-ZDHHC17 binding could mostly be attributed to ZDHHC17 residues Met-144, Lys-145, Asp-149, and Lys-179.

### ZDHHC17-MAP2K4-JNK/p38 Signaling Module Drives Malignant Progression in GBM

**Figure 6:**
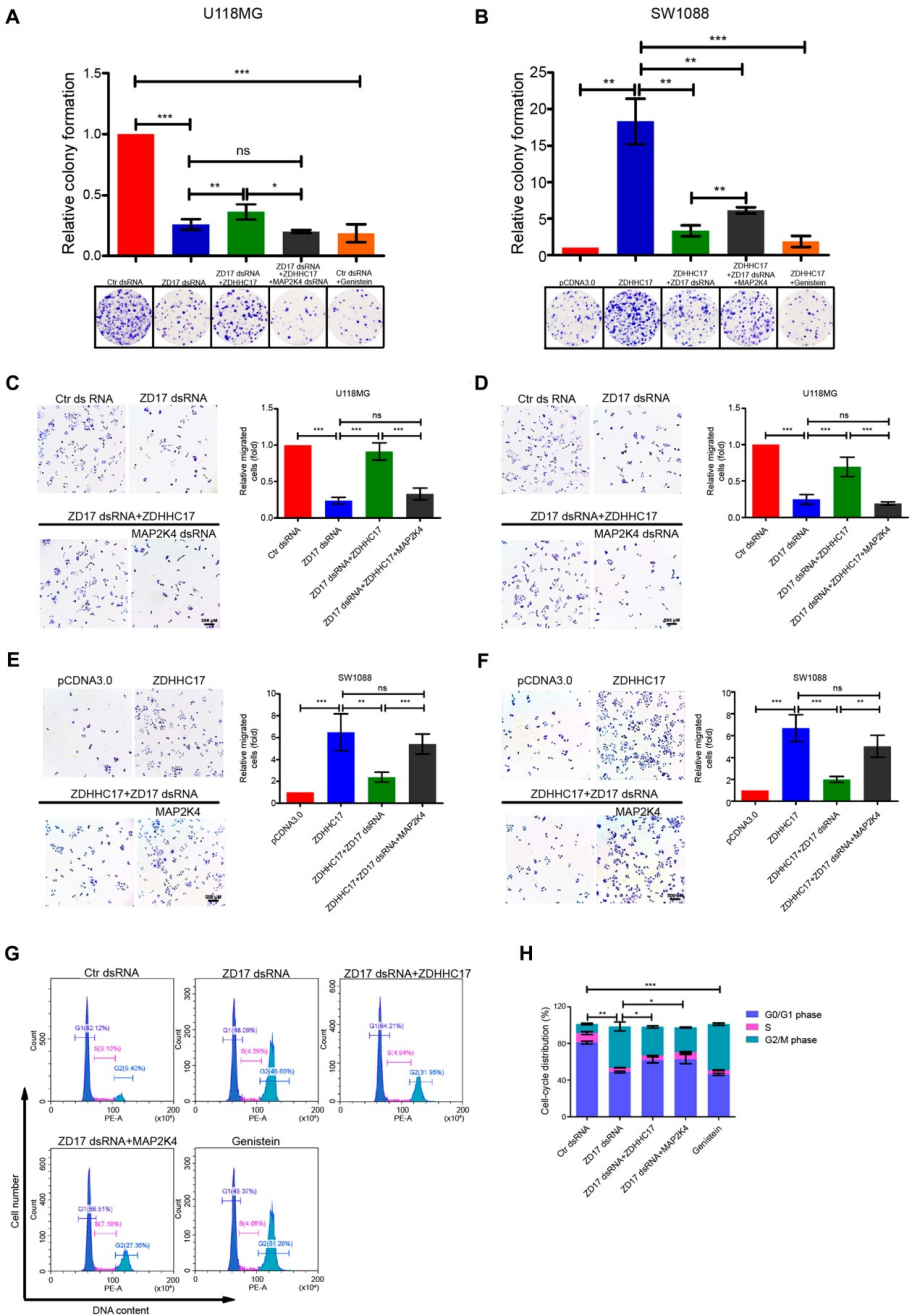
** ZDHHC17-MAP2K4 Signaling Module is Necessary for GBM Cell Tumorigenic and Invasive Phenotypes. (A, B)** Clonogenic survival of (A) U118MG cells transfected with control or ZDHHC17 dsRNA, further transfected with ZDHHC17 plasmid or MAP2K4 dsRNA, or treated with genistein (2.5 μM), and (B) SW1088 cells transfected with control or ZDHHC17 plasmid, further transfected with ZDHHC17 dsRNA or MAP2K4 plasmid, or genistein (2.5 μM) treated and ZDHHC17-expressing. Data represent the means ± SD from three separate experiments (*ns*, non-significant; *p < 0.05; **p < 0.01; ***p < 0.001, unpaired *t*-test). **(C-F)** Transwell analysis of (C, E) migratory and (D, F) invasive U118MG cells transfected with control or ZDHHC17 dsRNA, or further transfected with ZDHHC17 plasmid or MAP2K4 dsRNA (C, D), and SW1088 cells transfected with control or ZDHHC17 plasmid, or further transfected with ZDHHC17 dsRNA or MAP2K4 plasmid (E, F). Data represent the means ± SD from three separate experiments (*ns*, not significant; **p < 0.01; ***p < 0.001, unpaired *t*-test). Scale bar, 500 μm. **(G, H)** Cell cycle of U118MG cells transfected with control or ZDHHC17 dsRNA, further transfected with ZDHHC17 plasmid or MAP2K4 dsRNA, or genistein (2.5 μM)-treated, as detected by flow cytometry. The percent of cells in the G0-G1 phase, S-phase, and G2-M phase was calculated. Data represent the means ± SD from three separate experiments (*p < 0.05; **p < 0.01; ***p < 0.001, unpaired *t*-test).

Next, we assess the oncogenic function of the ZDHHC17-MAP2K4 signaling module in GBM. Foci formation frequency was significantly reduced in ZDHHC17-knockdown cells and was restored by subsequent ZDHHC17 overexpression (**Figure 6**A). MAP2K4 dsRNA transfection or genistein treatment also reduced ZDHHC17-mediated foci formation. These results were confirmed in patient-derived glioblastoma cells with ZDHHC17 overexpression (**Figure S10**A and **S10**B). The ZDHHC17-MAP2K4 signaling module could regulate the tumorigenic phenotype, whereas genistein could effectively suppress foci formation (**Figure 6**B).

Functional assays revealed that cell invasion and migration were related to ZDHHC17 and MAP2K4 expression levels, with genistein exhibiting a clearly inhibitory effect (**Figures 6**C-**6**F). These results were confirmed in patient-derived glioblastoma cells (**Figure S10**C-**S10**F). JNK or p38 agonists could partly rescue the down-regulation of migration, invasion, and foci formation by ZDHHC17 knockdown, whereas both p-JNK and p-p38 inhibitors could suppress the ZDHHC17-overexpressed tumorigenic and invasive phenotype (**Figures S11**A-**S11**C), demonstrating that these traits of ZDHHC17-expressing GBM cells are related to JNK and p38 activation.

More G2 phase but fewer G1 and S phase cells were detected after ZDHHC17-knockdown (**Figures 6**G and **6**H). MAP2K4 over-expression could rescue the cell cycle progression, as shown by the increased G1 and S phase, and reduced G2 phase cell counts. Consistently, genistein caused accumulation of the G2-phase cell subpopulation, suggesting that ZDHHC17 deficiency causes G2/M transition arrest. ZDHHC17 might regulate Golgi complex fragmentation via JNK2, thus leading to the G2/M transition (**Figures S12**).

**Figure 7:**
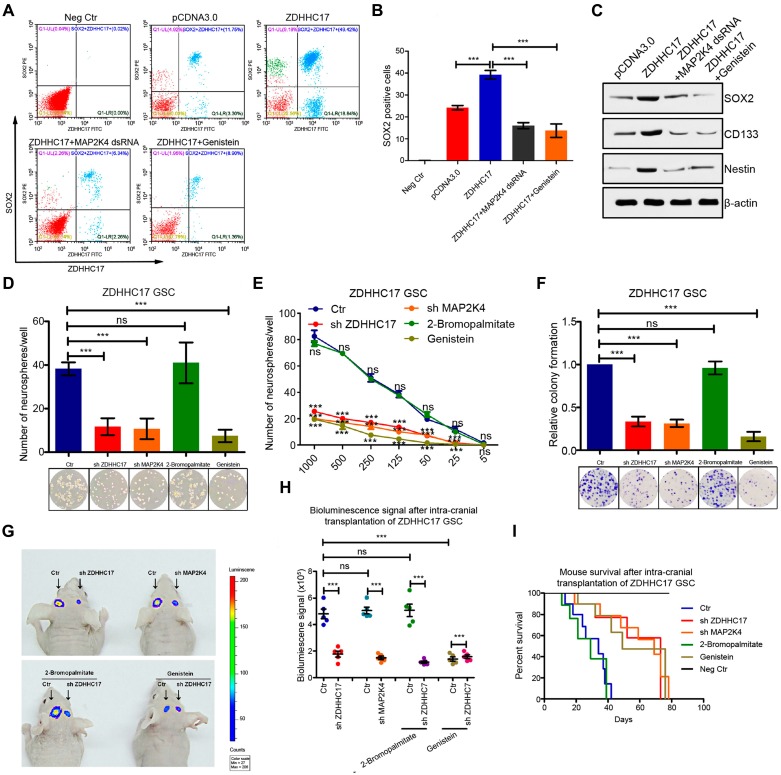
** ZDHHC17-MAP2K4 Signaling Module is Required for GBM Cell Neurosphere Formation. (A-C)** Flow cytometry and quantitation of SOX2 (stem cell marker) (A, B) and western blot of SOX2, CD133, and Nestin using β-actin as a loading control (C) in SW1088 cells transfected with ZDHHC17 plasmid or control, further transfected with MAP2K4 dsRNA, or genistein (2.5 μM)-treated. Data represent the means ± SD from three separate experiments (***p < 0.001, unpaired *t*-test). **(D)** Self-renewal of glioma stem cells (GSCs) originating from U118MG cells transfected with ZDHHC17 or MAP2K4 dsRNA, or genistein (2.5 μM) or 2-bromopalmitate (palmitoylation inhibitor; 200 μM)-treated, by neurosphere formation assay. Neurosphere numbers from dsRNA-transduced or inhibitor-treated ZDHHC17-expressing GSCs were calculated. Data represent the means ± SD from three separate experiments (*ns*, not significant; ***p < 0.001, unpaired *t*-test). **(E)** Neurosphere capacity by serial dilution assay of GSCs from U118MG cells transfected with ZDHHC17 or MAP2K4 dsRNA, or genistein (2.5 μM) or 2-bromopalmitate (200 μM)-treated. Data represent the means ± SD from three separate experiments (*ns,* not significant; ***p < 0.001, unpaired *t*-test). **(F)** Colonies formed by 200 viable GSCs from U118MG cells transfected with MAP2K4 dsRNA or ZDHHC17, or genistein (2.5 μM) or 2-bromopalmitate (200 μM)-treated. Data represent the means ± SD from three separate experiments (*ns*, not significant; ***p < 0.001, unpaired *t*-test). **(G)** Representative photon flux images from BALB/c mouse brains implanted with luciferase-expressing GSCs from U118MG cells transfected with control (left) or ZDHHC17 shRNA (right), then further genistein (50 mg kg^-1^ two days^-1^) or 2-bromopalmitate (100 mg kg^-1^ day^-1^)-treated. **(H)** Bioluminescence signal intensity in intracranial tumors between control (left) or ZDHHC17 shRNA (right) U118MG GSCs with or without 2-bromopalmitate or genistein (n = 5). Data represent the means ± SD (*ns*, not significant; *p < 0.05; **p < 0.01; ***p < 0.001, unpaired *t*-test). **(I)** Kaplan-Meier survival curve of animal survival following control or ZDHHC17 shRNA U118MG GSC cell injection with or without genistein or 2-bromopalmitate.

ZDHHC17 over-expression could help maintain glioma cell stemness because the upregulation of the SOX2-positive cell population, whereas MAP2K4 knockdown or genistein treatment suppressed glioma stem cell (GSC) self-renewal (**Figures 7**A and **7**B). As GSCs grew, the stemness markers cluster of differentiation CD133, Nestin, and SOX2 were also up-regulated by ZDHHC17 over-expression (**Figure 7**C). Notably, their expression was dependent on the ZDHHC17-MAP2K4 interaction module, as MAP2K4 knockdown or genistein treatment restored these protein levels to control values, or lower.

Cell lines with higher ZDHHC17 expression contained more clonogenic (neuro) sphere-forming cells (**Figure 7**D). However, GSC neurosphere formation capacity was reduced under free-floating neurosphere culture conditions following either ZDHHC17 or MAP2K4 knockdown. The patient-derived glioblastoma cells also displayed the ZDHHC17-MAP2K4-mediated modulation of GSCs self-renewal (Figure S9G and S9H). Besides, ZDHHC17-expressing GSCs transduced with control shRNA yielded markedly greater numbers of neurospheres at every dilution tested compared to the ZDHHC17- or MAP2K4- deficient GSCs (**Figure 7**E). These results suggested that the ZDHHC17-MAP2K4 signaling module is needed for GSC maintenance. Furthermore, loss of MAP2K4 or ZDHHC17, or genistein treatment decreased the number of colonies formed by GSCs, indicating reduced oncogenic potential (**Figure 7**F). Notably, the ZDHHC17-mediated GSC self-renewal showed a preference for JNK activation, as the p38 agonist barely restored the ZDHHC17 depletion-mediated inhibition of GSC maintenance, and the p-p38 inhibitor could not inhibit GSC self-renewal (**Figure S11**D).

To assess the link between ZDHHC17-MAP2K4 signaling module and GBM tumorigenesis, ZDHHC17-expressing and -deficient GSCs derived from luciferase-expressing U118MG cells were implanted into the brains of immunocompromised NOD/SCID mice (**Figures 7**G and **7**H). ZDHHC17-expressing GSCs could efficiently develop and form intracranial tumors *in vivo*, whereas ZDHHC17 depletion clearly suppressed tumor growth. Consistent with these results, animal death was within 38-42 days after ZDHHC17-expressing GSC injection (**Figure 7**I), whereas MAP2K4 knockdown or genistein injection inhibited tumor growth and extended the survival of animals implanted with ZDHHC17-expressing cells. The patient-derived xenograft (PDX) mice also revealed the ZDHHC17-MAP2K4 signaling module could obviously regulate the tumorigenic phenotype, and genistein injection inhibited tumor growth (**Figure S10**I and **S10**J).

### ZDHHC17-MAP2K4 Signaling Module Promotes Chemoradiotherapy Resistance in Glioma

**Figure 8:**
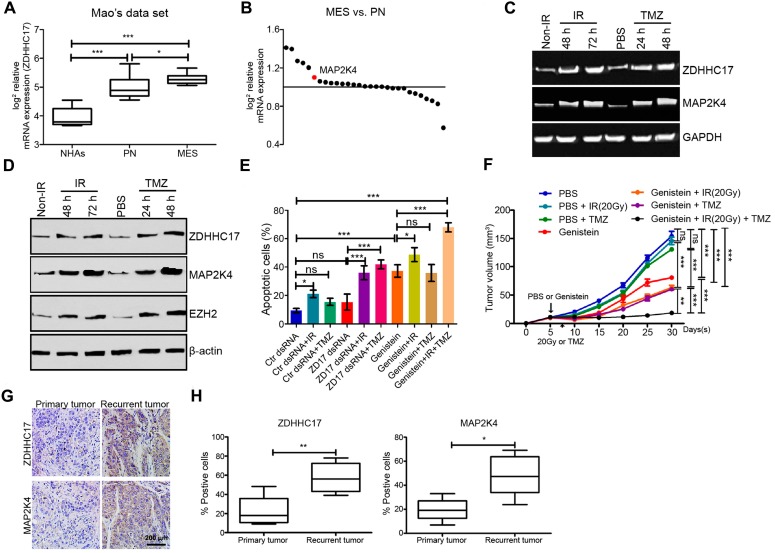
** ZDHHC17-MAP2K4 Signaling Module Promotes Chemoradiotherapy Resistance in GBM Spheres. (A)** mRNA expression analysis (Mao's dataset, GSE67089) of *ZDHHC17* expression in mesenchymal (MES) GSCs compared to normal astrocytes, or proneural (PN) GSCs. **(B)** Genome-wide transcriptome microarray analysis (GSE67089) of MAPKKs showing MAP2K4 up-regulation in MES compared with PN GSCs. **(C)** RT-PCR for *ZDHHC17* and *MAP2K4* in post-radiation GSCs from U118MG (6 Gy) or temozolomide (TMZ)-treated (25 μM) versus naive GSCs. **(D)** Western blot for ZDHHC17, MAP2K4, and EZH2 in post-radiation GSCs from U118MG (6 Gy) or TMZ-treated GSCs (25 μM) versus naive GSCs. **(E)** Flow cytometric analysis for apoptosis in GSCs pre-transduced with control or ZDHHC17 dsRNA, then treated with or without genistein (2.5 μM), radiation (6 Gy), and TMZ (25 μM). **(F)** BALB/c mice were subcutaneously injected with GSCs from U118MG. After five days, the nude mice were treated with 20 Gy X-irradiation (4.5-4.6 Gy/min), TMZ (50 mg kg^-1^ two days^-1^, gastric infusion), or genistein (100 mg/kg daily, tail vein injection). Tumor weight was quantified. Data represent the means ± SD from five separate experiments (*ns*, not significant; **p < 0.01; ***p < 0.001, unpaired *t*-test). **(G)** Typical immunohistochemistry images for ZDHHC17 and MAP2K4 in primary untreated and post-radiation and -TMZ chemotherapy recurrent tumors from matched patients with GBM. Scale bars, 200 µm. **(H)** Percentage of ZDHHC17 or MAP2K4 positive-stained cells in primary untreated and recurrent tumors from matched patients with GBM (n = 5; *p < 0.05; **p < 0.01; ***p < 0.001).

Next, we investigated whether the ZDHHC17-MAP2K4 signaling module contributes to chemo/radio-therapy resistance in glioma, thereby leading to malignant progression and tumor recurrence after therapeutic failure. ZDHHC17 (**Figure 8**A) and MAP2K4 (**Figure 8**B) expression was markedly enhanced in mesenchymal GSCs, which are closely associated with post-radiation or chemotherapeutic glioma recurrence 25. Both western blotting and RT-PCR results indicated that MAP2K4 and ZDHHC17 were increased in the glioma spheres after 6 Gy radiation or TMZ treatment, similar to EZH2 expression (**Figures 8**C and **8**D). Glioma cell apoptosis was then detected using ZDHHC17 dsRNA-transduced glioma spheres, with or without radiation (6 Gy) or TMZ treatment (**Figure 8**E). Apoptotic cell proportions were apparently raised after TMZ treatment or radiation when combined with ZDHHC17 knockdown, compared to those from TMZ treatment or radiation alone. Moreover, genistein presented a more effective treatment prospect; as it dramatically increased glioma cell apoptosis after radiation or TMZ treatment, even if used alone. The* in vivo* results also showed that genistein effectively inhibited tumor growth accompanied by chemoradiotherapy in a subcutaneous transplantation glioma model (**Figure 8**F).

To investigate whether these results were clinically relevant, five matched glioma tissues were collected from the initial surgical treatment for untreated tumors and from the second surgical treatment after radiation and TMZ chemotherapy treatment failure for ZDHHC17 and MAP2K4 IHC analysis. Although ZDHHC17 and MAP2K4 staining intensities varied among the untreated glioma tissues, ZDHHC17 and MAP2K4 expression tended to increase in recurrent compared to naive tumor sections (4 of 5) (**Figure 8**G). Similarly, MAP2K4- or ZDHHC17- positive tumor cells or proportions were raised overall in recurrent compared with matched primary tumors (**Figure 8**H). Collectively, these findings indicate that the ZDHHC17-MAP2K4 signaling module is activated in post-radiation or chemotherapeutic glioma cells or tissues, suggesting an important role in the development of chemoradiotherapy resistance.

## Discussion

Dysregulation of DHHC PAT activity is associated with various diseases including Huntington's disease, schizophrenia, and X-linked mental retardation; however, the DHHC protein family and their substrates may also play a significant role in tumorigenesis 10, 26, 27. In addition to PAT- mediated protein palmitoylation, we demonstrate here that ZDHHC17 also plays an apparently unique role in the PAT protein family by interacting with MAP2K4 via the N-terminal signaling and protein-protein interaction ankyrin domain, to activate JNK/p38 and regulate GBM malignant development and progression. By manipulating this specific ZDHHC17-MAP2K4 interaction, we identified genistein as a candidate inhibitor for the ZDHHC17-MAP2K4 signaling module, and provide a potential therapeutic strategy for GBM patients.

DHHC PATs can predominantly exhibit either oncogenic or tumor suppressor function in specific cancers; e.g., ZDHHC2 as a tumor suppressor in breast, lung, and prostate tumors 28-30. Similarly ZDHHC3 deletions are found in kidney renal clear cell carcinoma and ZDHHC7 is deleted in 10% of prostate adenocarcinoma as well as in breast and ovarian cancer 31, 32, whereas p53-mutant glioma shows ZDHHC5 up-regulation 16. However, the same ZDHHC gene may be amplified in some tumor types but deleted in others (e.g. ZDHHC17, ZDHHC20, and ZDHHC21). ZDHHC17 may also function PAT-independently in some cellular contexts. ZDHHC17 is up-regulated in stomach, colon, brain, and lung tumors concordant with tumor stage gradation 33, 34, but down-regulated in liver, pancreas, thyroid gland, and vulva cancers. The apparent contradictory roles in cancer may result from small sample numbers or cell type and context-specific substrates. Our *ex vivo* and* in vivo* assays suggest that ZDHHC17 is a binding partner of JNK/p38. MAP2K4, a JNK/p38 activator and possible oncogenic driver 7, 35, is likewise selectively and functionally connected with ZDHHC17, whereas the JNK activator MAP2K7 showed minimal ZDHHC17 interaction and could not rescue ZDHHC17 knockdown-mediated tumorigenic GSC phenotypes upon over-expression (**Figure S13**). Both ZDHHC17 and MAP2K4 are elevated in early embryonic cortical neural precursor cells, suggesting relationship to an undifferentiated or stemness state. Moreover, *MAP2K4* localization near the tumor suppressor p53 on chromosome 17 implied roles in glioma initiation and malignancy 36, 37. Here, we disclosed that ZDHHC17 over-expression and mutations in KRAS, TERT, and p53 oncogenes in human neural stem cells was sufficient for fully malignant and rapid transformation. In addition, MAP2K4 played a significant function in this process, with MAP2K4 deficiency suppressing TERT/CA-KRAS/DN-p53 glioma cell tumorigenicity. Further studies implied that MAP2K4-ZDHHC17-JNK/p38 forms a signaling module for JNK/p38 activation in glioma, including gliomagenesis and malignant progression. Notably, ZDHHC17-promoted activation of JNK/p38 was PAT-independent, and ZDHHC17-expressing glioma cell malignancy characteristics were not suppressed by the PAT inhibitor, 2-bromopalmitate (2BP). The observation of varied ZDHHC17 substrate palmitoylation and interaction profiles, along with JNK or p38 pathway activation, in several neurological diseases 38 implies that different ZDHHC17 functions are systematically linked and may directly or indirectly contribute to neurological disease pathogenesis.

JNK/p38 signaling is implicated in cancer initiation, progression, metastasis, and chemotherapy response 4, 39-41 and elevated p38 activity was observed in a glioblastoma cell line panel and human glioma samples 4. c-Jun and JNK phosphorylation similarly significantly correlates with glioma histological grade 8, 42. Thus, JNK/p38 MAPKs are considered as promising drug targets for cancer therapy. However, the mechanisms regulating JNK/p38 activation in glioma are poorly understood. Our results indicated that ZDHHC17 recruits JNK/p38 and MAP2K4 to form a signaling module for JNK/p38 activation during glioma progression and malignant development. This is consistent with the suppression of soft-agar colony and sphere formation in ZDHHC17-expressing glioma cells upon pharmacological inhibition of JNK1/2 (p38) or JNK-IN-8 (PH-797804) Moreover, JNK1/2 (anisomycin) or p38 (P79350) activation partly rescued the ZDHHC17 knockdown-mediated migration, invasion, and foci formation down-regulation.

JNK or p38 also function independently in ZDHHC17-expressing glioma cells. JNKs have a role in maintaining GSC properties, which underlie poor prognosis of patients with glioblastoma. Here, we found that ZDHHC17-mediated GSC self-renewal exhibited a preference for JNK activation, as a p38 agonist or *p*-p38 inhibitor did not respectively counteract ZDHHC17 depletion-induced disruption of GSC maintenance or inhibit GSC self-renewal. ZDHHC17 largely localizes to the Golgi, and its deficiency led to G2/M transition arrest, along with increased proportion of cells with dense Golgi. The latter was also observed after JNK2 inhibition in ZDHHC17-overexpressing cells, as JNK2 is needed for GRASP65-Ser277 phosphorylation, which is required for Golgi fragmentation during the late G2 phase and for G2/M transition 43. Thus, our findings establish a causal link between the ZDHHC17-JNK2-mediated Golgi complex fragmentation and cell cycle progression.

Although PATs, as transmembrane S-acyltransferases, contain a conserved zinc finger DHHC (DHHC) domain 10, 44, few PAT inhibitors, especially DHHC-isoform-specific inhibitors, are available. The well-established lipid-based 2BP is a nonspecific inhibitor, blocking palmitoyltransferase activity of all DHHCs tested 45, 46, raising the potential for unknown side effects. Inhibitor selectivity may be achieved by exploiting the structural heterogeneity in C- and the N- terminal domains among the isoforms. Alone among 23 DHHC enzymes, ZDHHC17 has an ankyrin repeat domain at its cytosolic N terminus 47, 48. The ANK domains of DHHC enzymes are predicted to engage in numerous interactions and facilitate both S-acylation-independent functions and substrate recruitment 47. ZDHHC17 interacts with MAP2K4 via its ANK domain to activate JNK/p38 and regulate malignant GBM development and progression. By manipulating this specific interaction, we identified genistein as a candidate inhibitor for the ZDHHC17-MAP2K4 signaling module. Notably, the genistein binding-domain for ZDHHC17 not only provides the main binding affinity between ZDHHC17 and MAP2K4, but is also the interaction domain for MAP2K4 PKc kinase domain binding. Thus these results support the hypothesis that genistein directly blocks the ZDHHC17 and MAP2K4 interaction and inhibits ZDHHC17-MAP2K4 kinase activity.

Genistein thus may provide a potential therapeutic strategy for patients with ZDHHC17-expressing glioma. As predicted, our phase II trial revealed the good therapeutic effect of genistein in this cohort (unpublished data). Overall, 12 patients (40%) completed at the least one year of treatment without progression, of which 6 had WHO II disease at enrollment (20%). The treatment was discontinued for 8 patients during the first year without progression, and 3 patients remain on treatment with less than 1 year of follow-up. Kaplan-Meier survival analysis revealed that patients with genistein treatment had longer OS and DFS than the no treatment group. Of the 30 patients, 6 were still alive at the time of analysis.

Genistein produces many disparate or uncertain effects, such as inhibiting cell proliferation and restraining estrogenic activity in breast cancer, and inducing cell apoptosis and inhibiting cell invasion in prostate cancer 49, 50. These effects are concentration dependent with no less than micromolar concentrations. As the concentration increases, the effect specificity decreases. Although mechanistic studies in the above analyses show that genistein could inhibit MAPK activation, its pharmacological target for suppressing these biological roles is not elucidated. Here, we showed that genistein has an IC_50_ value in the nano-molar range for the ZDHHC17-MAP2K4 interaction and specifically suppresses glioma progression and malignant development. To enhance its safety and effectiveness, future studies should focus on improving its bioavailability and developing more efficacious derivatives.

Overall, the present study showed that ZDHHC17 was up-regulated in glioma as compared to normal brain tissue, and that it recruited MAP2K4 and JNK/p38 to build a signaling module for JNK/p38 activation in GBM. The ZDHHC17-MAP2K4-JNK/p38 signaling module was shown to contribute to GBM malignant progression and development by promoting glioma cell tumorigenicity and GSC self-renewal. In addition, genistein was identified as the lead candidate for glioma therapy as a specific ZDHHC17-MAP2K4 inhibitor. Notably, we further revealed that genistein treatment, compared to no treatment, had good therapeutic effect in patients with ZDHHC17- expressing glioma.

## Supplementary Material

Supplementary figures and tables.

## Abbreviations

DFS: disease-free survival
ERK: extracellular regulated protein kinase
GBM: Glioblastoma Multiforme
GSC: glioma stem cell
hNSC: human neural stem cell
IC_50_: Inhibitory concentration 50
IHC: immunohistochemistry
JNK: c-Jun N-terminal kinase
MAPK: the mitogen-activated protein kinase
MAP2K4: mitogen-activated protein kinase kinase 4
OS: overall survival
PAT: palmitoyl-acyltransferases
TMZ: temozolomide
ZDHHC17: Zinc-finger DHHC-type containing 17
