# Supplementary material for: Activation of JNK and p38 MAPK Mediated by ZDHHC17 Drives Glioblastoma Multiforme Development and Malignant Progression
Source: Theranostics. 2020 Jan 1;10(3):998–1015. doi: 10.7150/thno.40076 (PMC6956818; doi:10.7150/thno.40076)
Supplement: Supplementary file 1 — Supplementary figures and tables. [file thnov10p0998s1.pdf]

Supplementary Figure 1

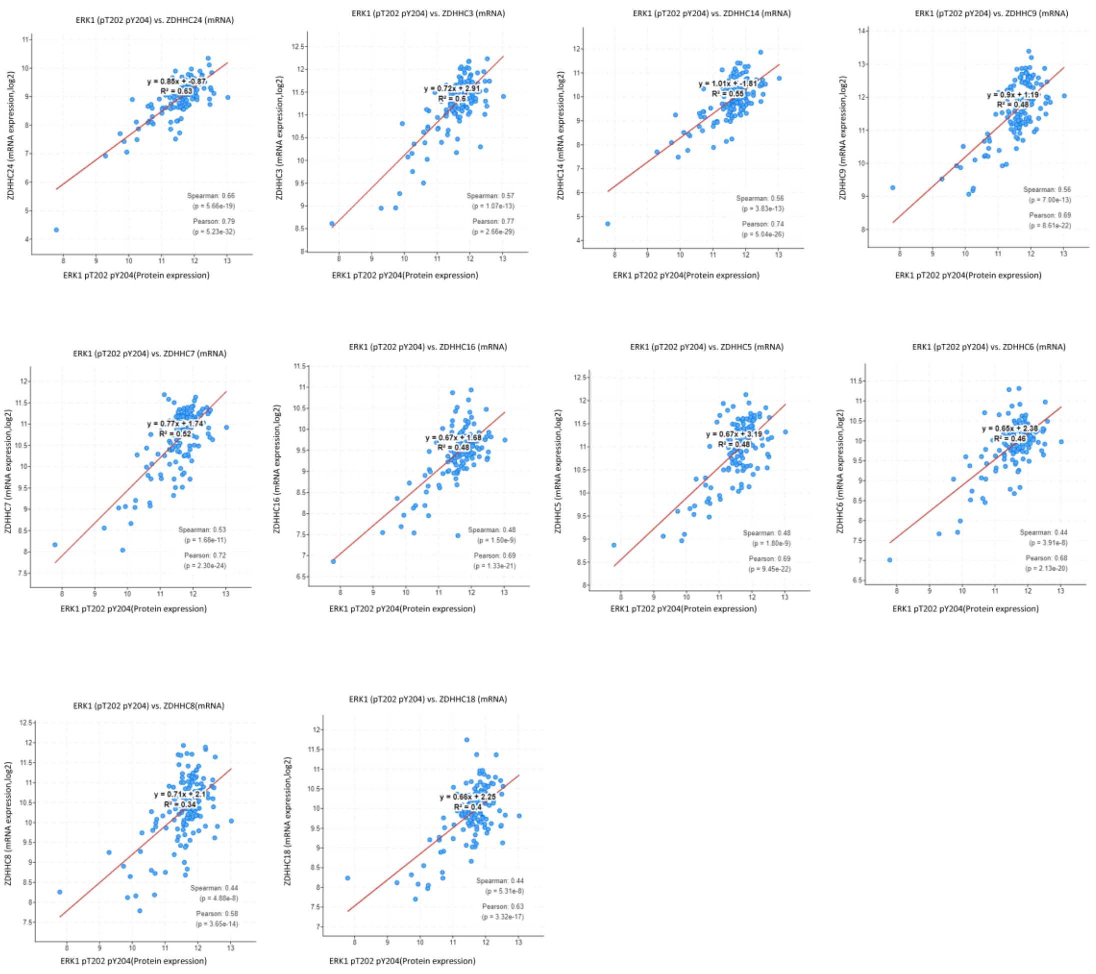

Supplementary Figure 1. ERK1(pT202, pY204; Protein Expression) Co-expression with different DHHCs (TOP10; mRNA Expression) in Glioblastoma Multiforme (GBM) using The Cancer Genome Atlas (TCGA) Database

Supplementary Figure 2

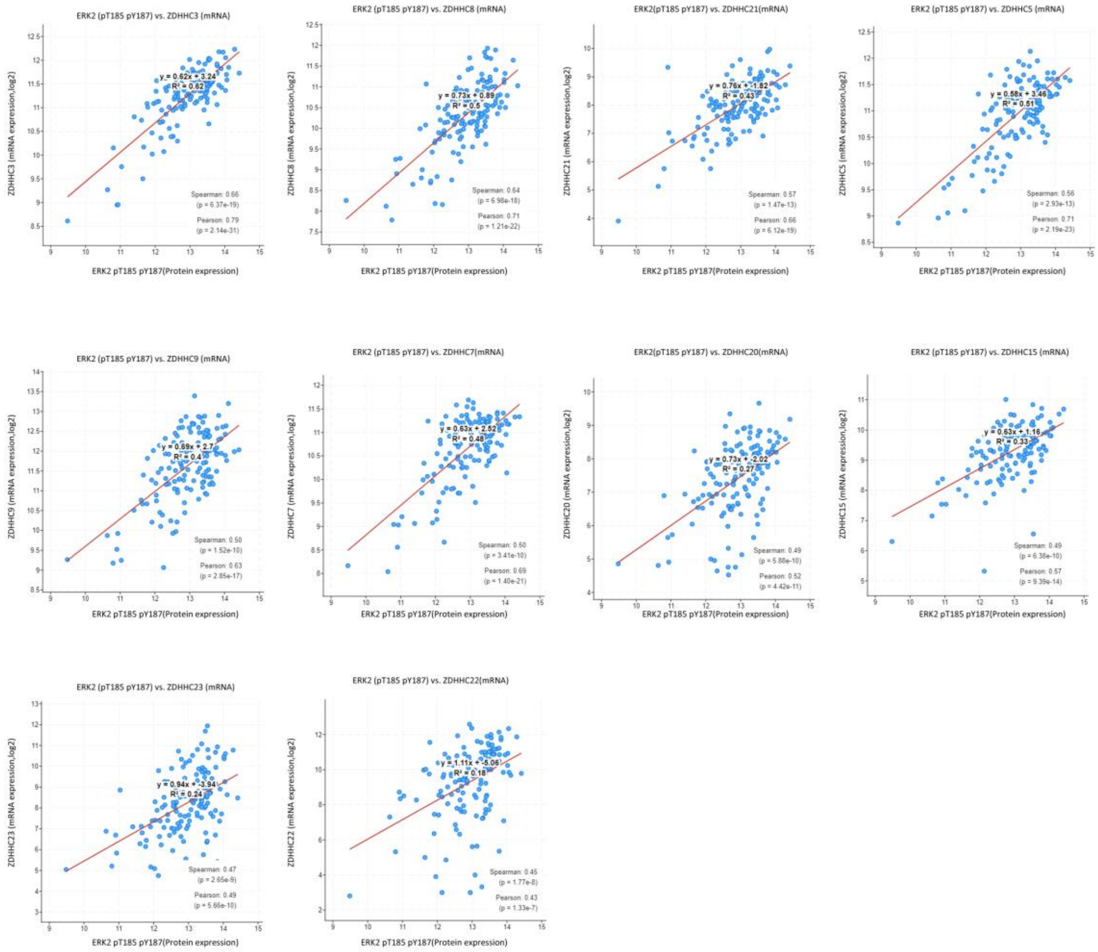

Supplementary Figure 2. ERK2(pT185, pY187; Protein Expression) Co-expression with different DHHCs (TOP10; mRNA Expression) in GBM using TCGA Database

Supplementary Figure 3

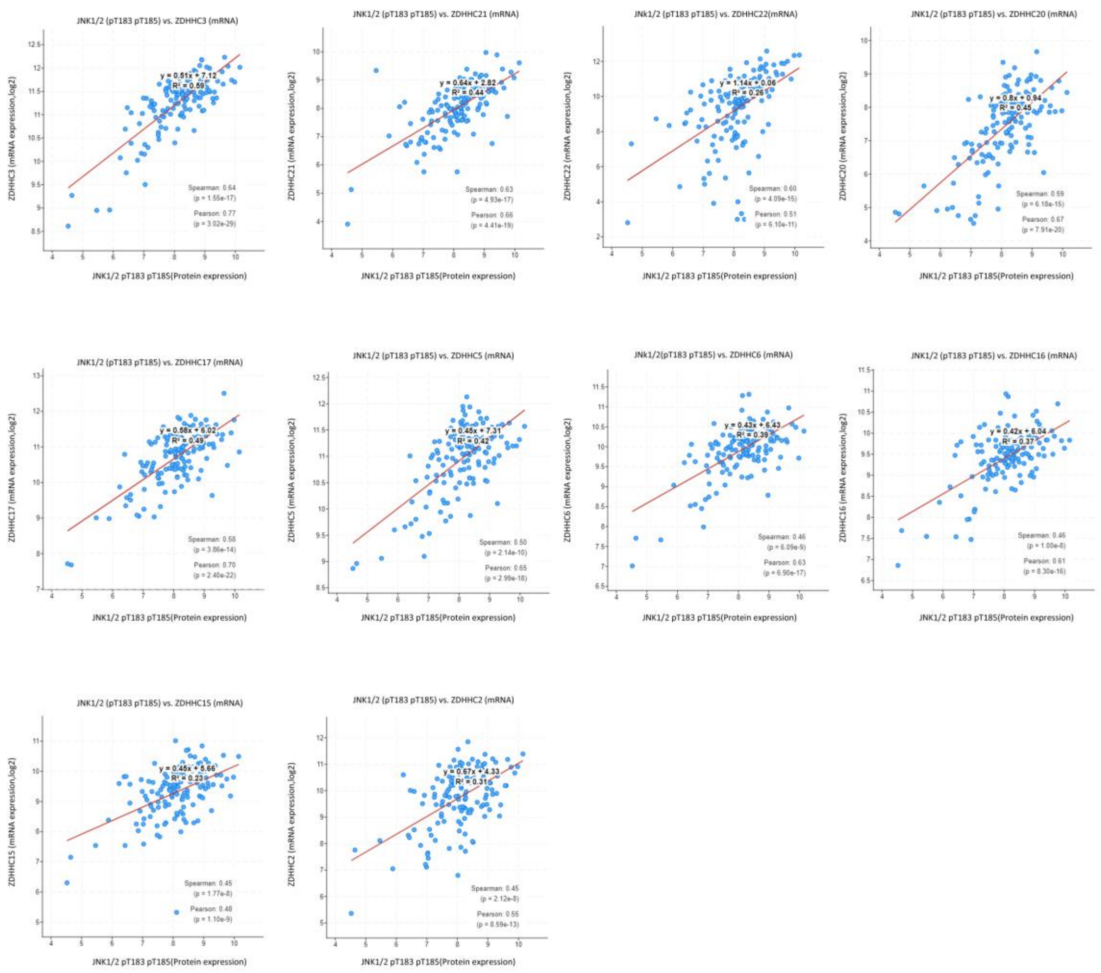

Supplementary Figure 3. JNK1/2(pT183, pT185; Protein Expression) Co-expression with different DHHCs (TOP10; mRNA Expression) in GBM using TCGA Database

Supplementary Figure 4

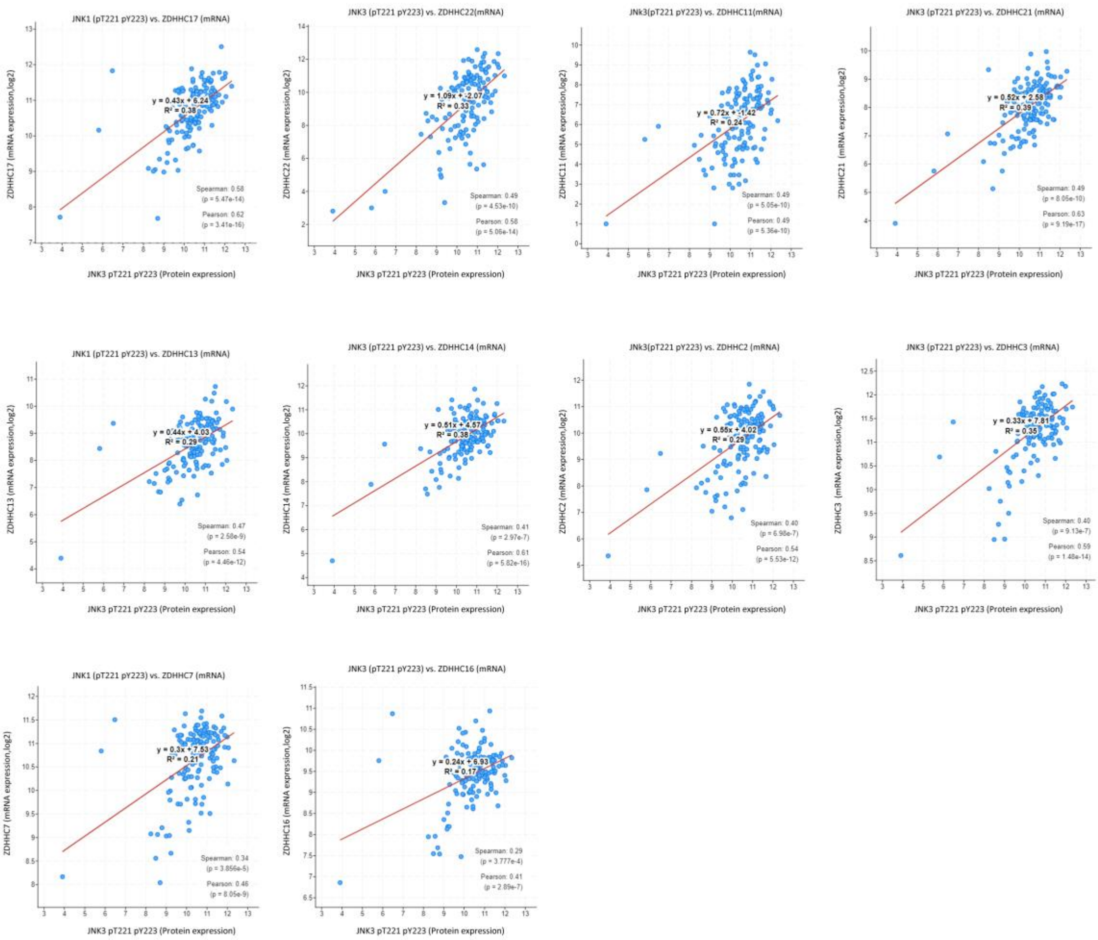

Supplementary Figure 4. JNK3(pT221, pY223; Protein Expression) Co-expression with different DHHCs (TOP10; mRNA Expression) in GBM using TCGA Database

Supplementary Figure 5

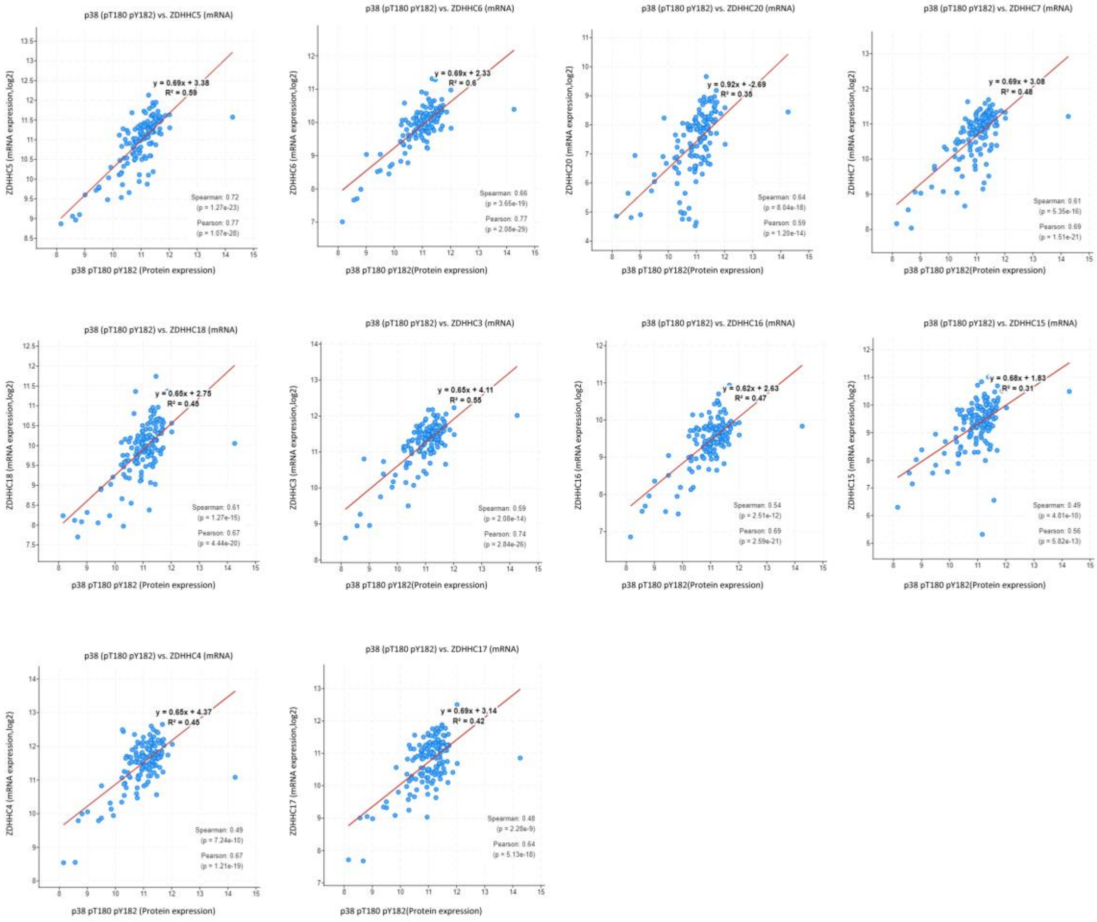

Supplementary Figure 5. p38(pT180, pY182; Protein Expression) Co-expression with different DHHCs (TOP10; mRNA Expression) in GBM using TCGA Database

Supplementary Figure 6

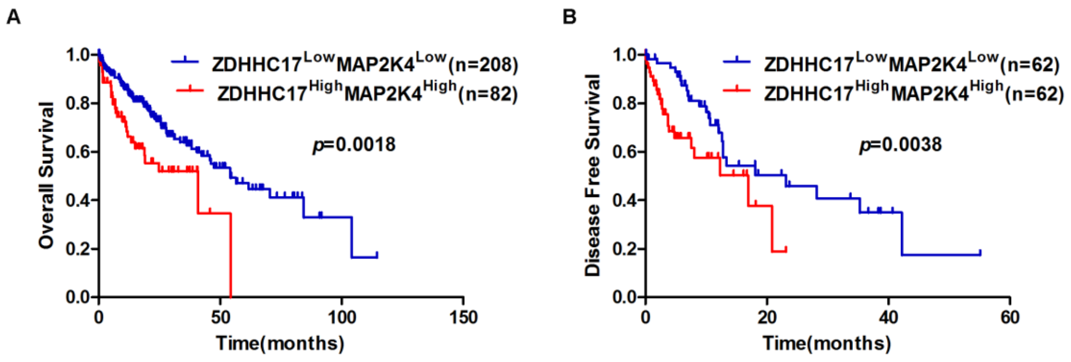

Supplementary Figure 6. Patient Survival is Associated with Low/High ZDHHC17-MAP2K4 Co-expression

(A, B) Cumulative overall survival (A) and disease-free survival (B) of patients with GBM with low/high ZDHHC17-MAP2K4 co-expression levels (based on median ZDHHC17 and MAP2K4 levels, respectively) estimated using the Kaplan-Meier method and compared with the log-rank test in the same set of patients (**$p < 0.01$).

Supplementary Figure 7

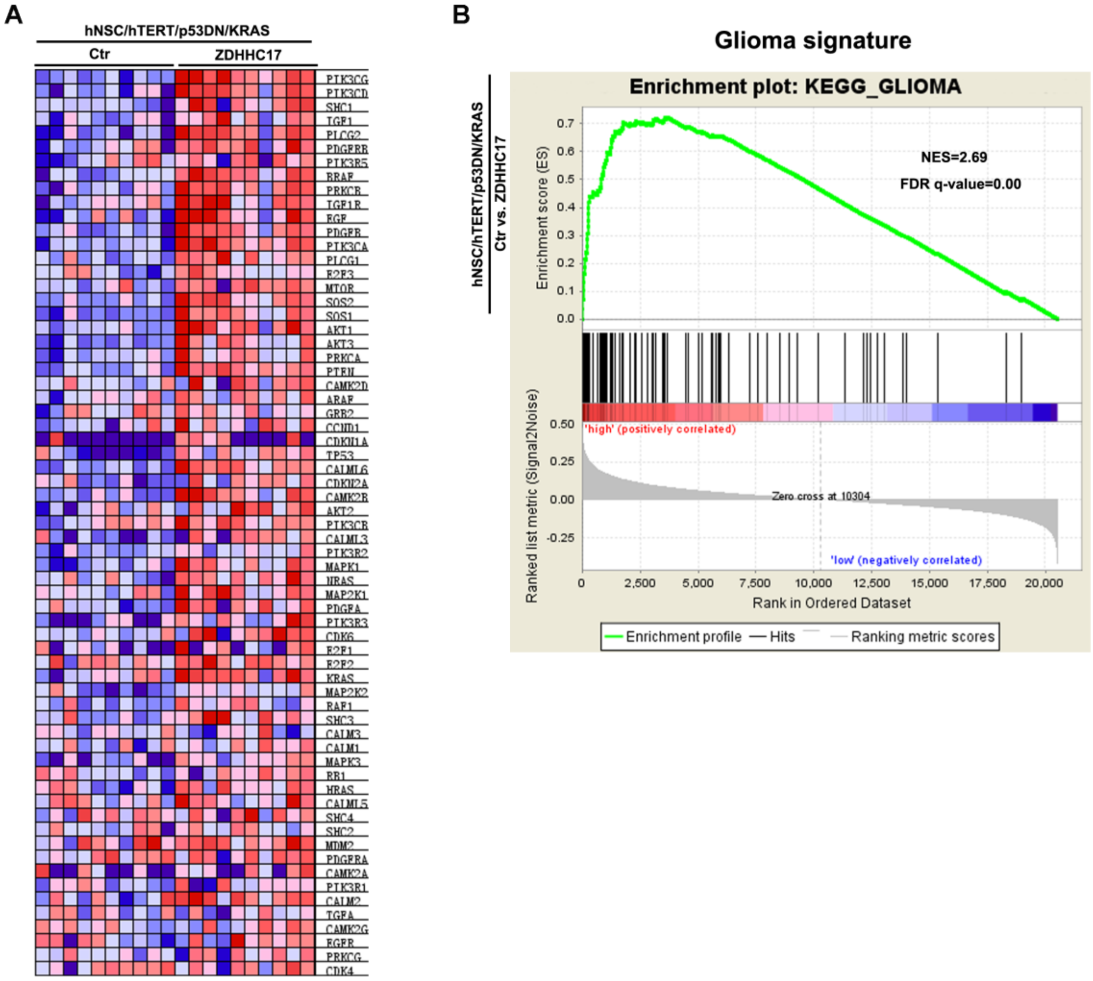

Supplementary Figure 7. hNSCs Harboring TERT/CA-KRAS/DN-p53/ZDHHC17 Exhibit a More Obvious Glioma Signature than Control hNSCs

(A) Hierarchical clustering using complete linkage of the gene expression associated with glioma features profiles among hNSCs harboring TERT/CA-KRAS/DN-p53/ZDHHC17 and wild-type control.

(B) Gene Set Enrichment Analysis (GSEA) highlighting positive association of increased *ZDHHC17* expression levels with glioma signature. FDR = false discovery rate; NES = normalized enrichment score.

Supplementary Figure 8

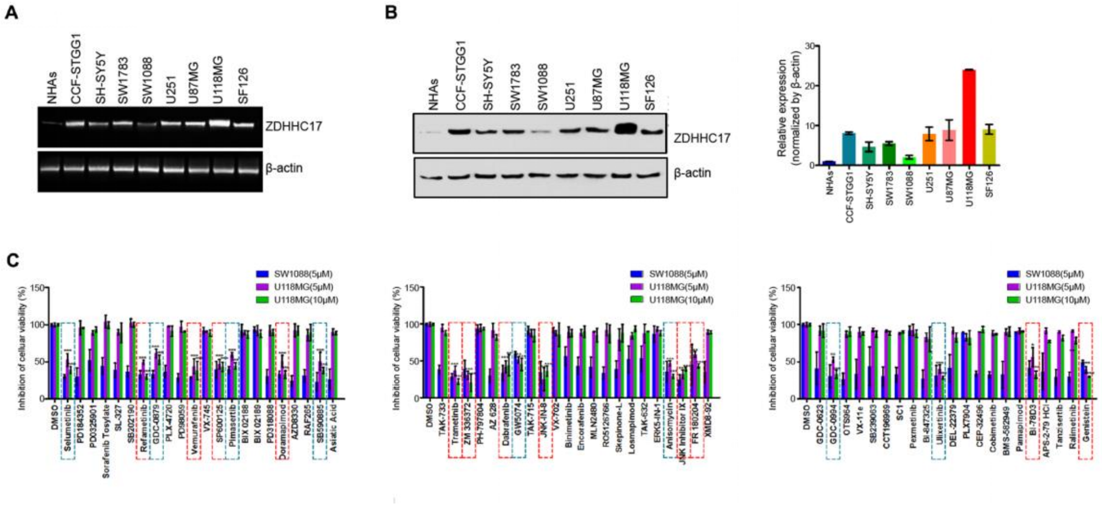

Supplementary Figure 8. Genistein could effectively Inhibit the ZDHHC17-expressed GBM Cell Growth.

(A, B) ZDHHC17 mRNA levels and protein accumulation in normal human astrocytes (NHAs) and GBM cell lines tested by RT-PCR (A) and western blot (B). β-actin was used as a loading control.

(C) SW1088 and U118MG cell viability after treatment with indicated concentrations of indicated inhibitors from the MAPK Compound Library. Data represent the means ± SD from three separate experiments (**$p < 0.01$; ***$p < 0.001$, unpaired *t*-test).

Supplementary Figure 9

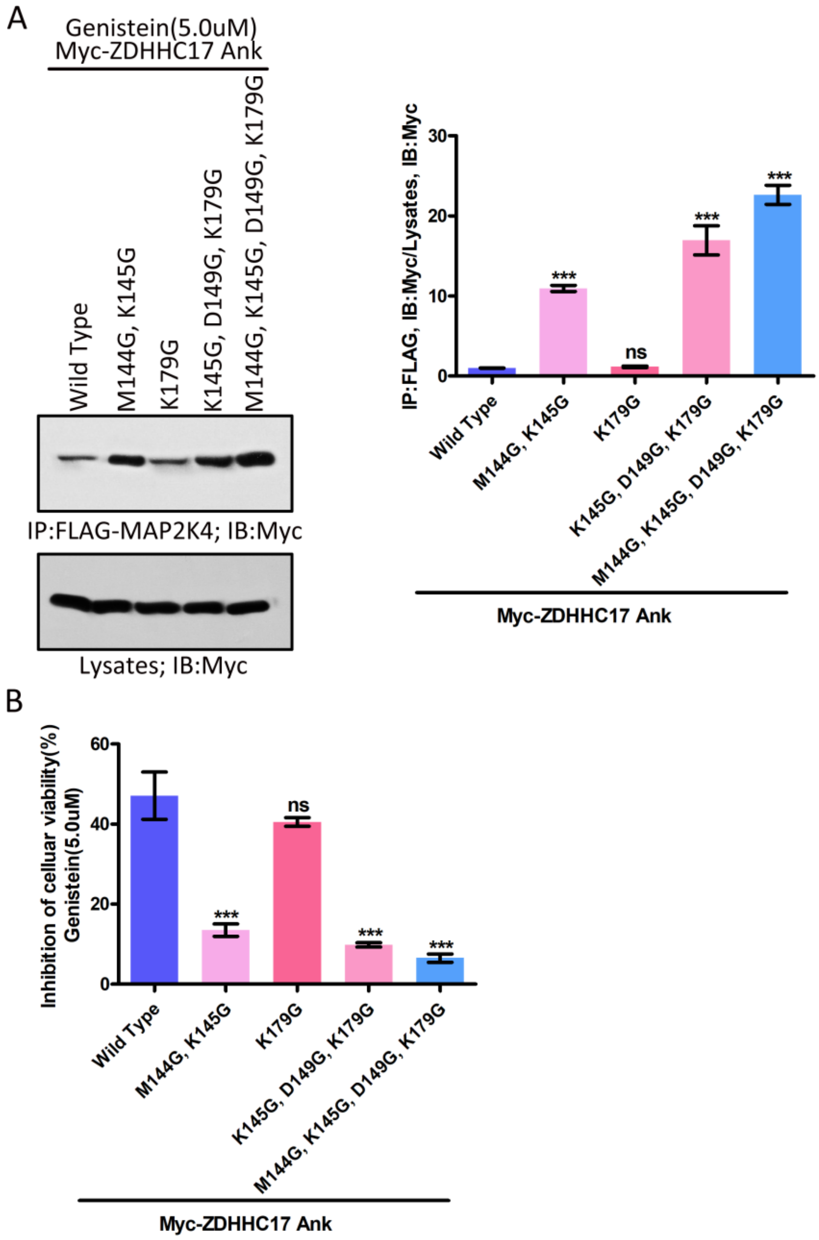

Supplementary Figure 9. Specific Residues for the Genistein-ZDHHC17 Binding

(A) IP of lysates from HEK293 cells expressing Flag-MAP2K4 and Myc-ZDHHC17 ANK mutants, treated with gensitein (5.0 μM), and followed by IB with anti-Flag antibodies and anti-Myc. Data represent the means ± SD from three separate experiments (*ns*, not significant; **p* < 0.05; ***p* <0.01; ****p* < 0.001, unpaired *t* test).

(B) Cell viabilities of U118MG cells were stably transfected with Myc-ZDHHC17 ANK mutants, and treated with genstein (5.0 μM).

Supplementary Figure 10

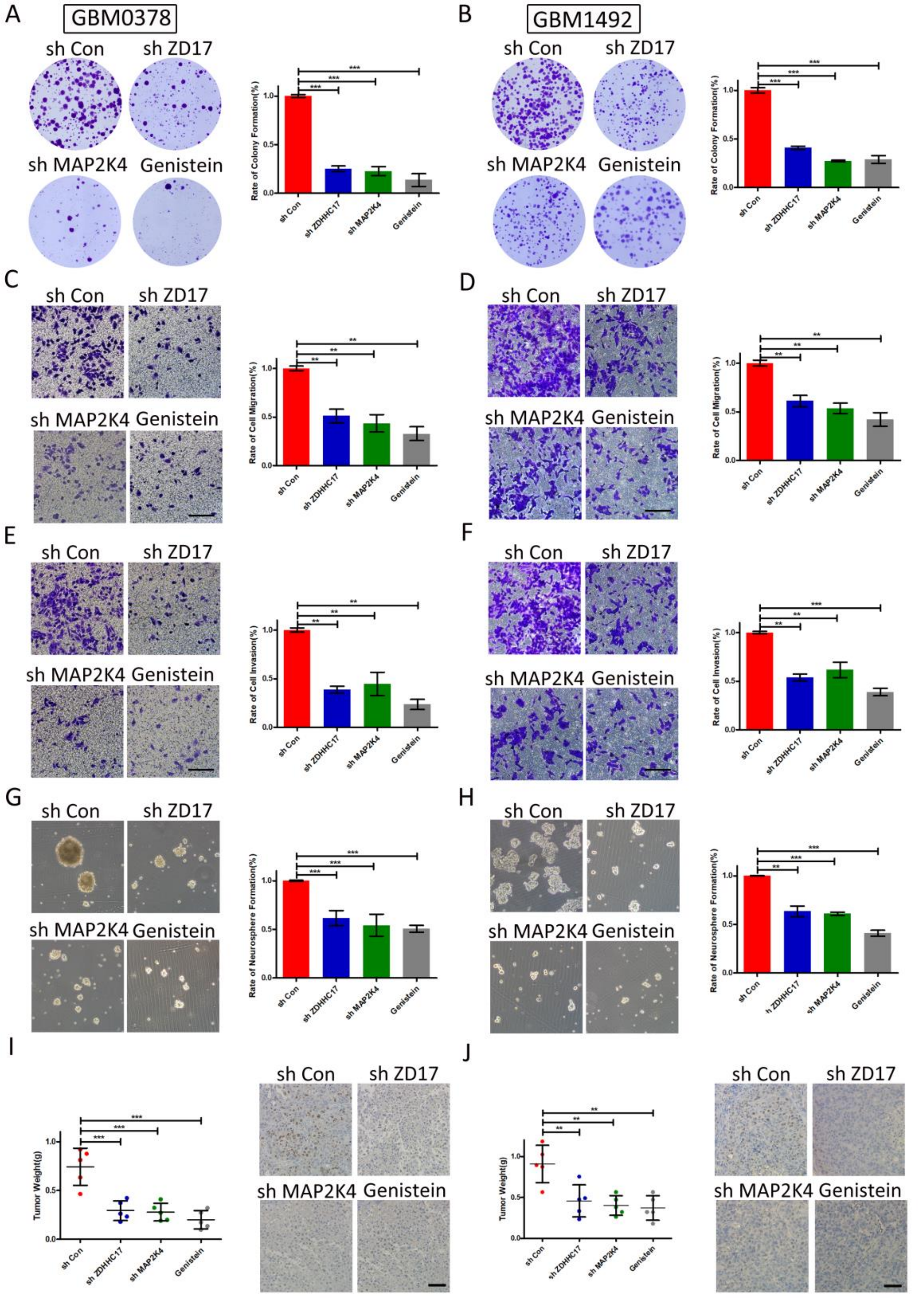

Supplementary Figure 10. ZDHHC17-MAP2K4 Signaling Module is Necessary for Tumorigenic and Invasive Phenotypes in Patient-derived Glioblastoma Cells

(A, B) Clonogenic survival of (A) GBM0378 cells or (B) GBM1492 cells stably transfected with

control, ZDHHC17 shRNA, or MAP2K4 shRNA, or treated with genistein (2.5 μM). Data represent the means ± SD from three separate experiments (*ns*, non significant; $*p < 0.05$; $**p < 0.01$; $***p < 0.001$, unpaired *t* test).

(C–F) Transwell analysis of (C, E) migratory and (D, F) invasive (C, D) GBM0378 cells or (E, F) GBM1492 cells stably transfected with control, ZDHHC17 shRNA, or MAP2K4 shRNA, or treated with genistein (2.5 μM). Data represent the means ± SD from three separate experiments (*ns*, not significant; $**p < 0.01$; $***p < 0.001$, unpaired *t* test). Scale bar, 500 μm.

(G, H) Colonies formed by 200 viable GSCs from (G) GBM0378 cells or (H) GBM1492 cells stably transfected with control, ZDHHC17 shRNA, or MAP2K4 shRNA, or treated with genistein (2.5 μM). Data represent the means ± SD from three separate experiments (*ns*, not significant; $***p < 0.001$, unpaired *t* test).

(I, J) Tumor weight of (I) GBM0378 and (J) GBM1492 PDX mice treated with control, ZDHHC17 shRNA, MAP2K4 shRNA -expressing lentivirus or genstein (n = 5; $**p < 0.01$; $***p < 0.001$). Percentage of Ki-67 positive-stained cells in tumor of PDX mice. Scale bar, 200 μm.

Supplementary Figure 11

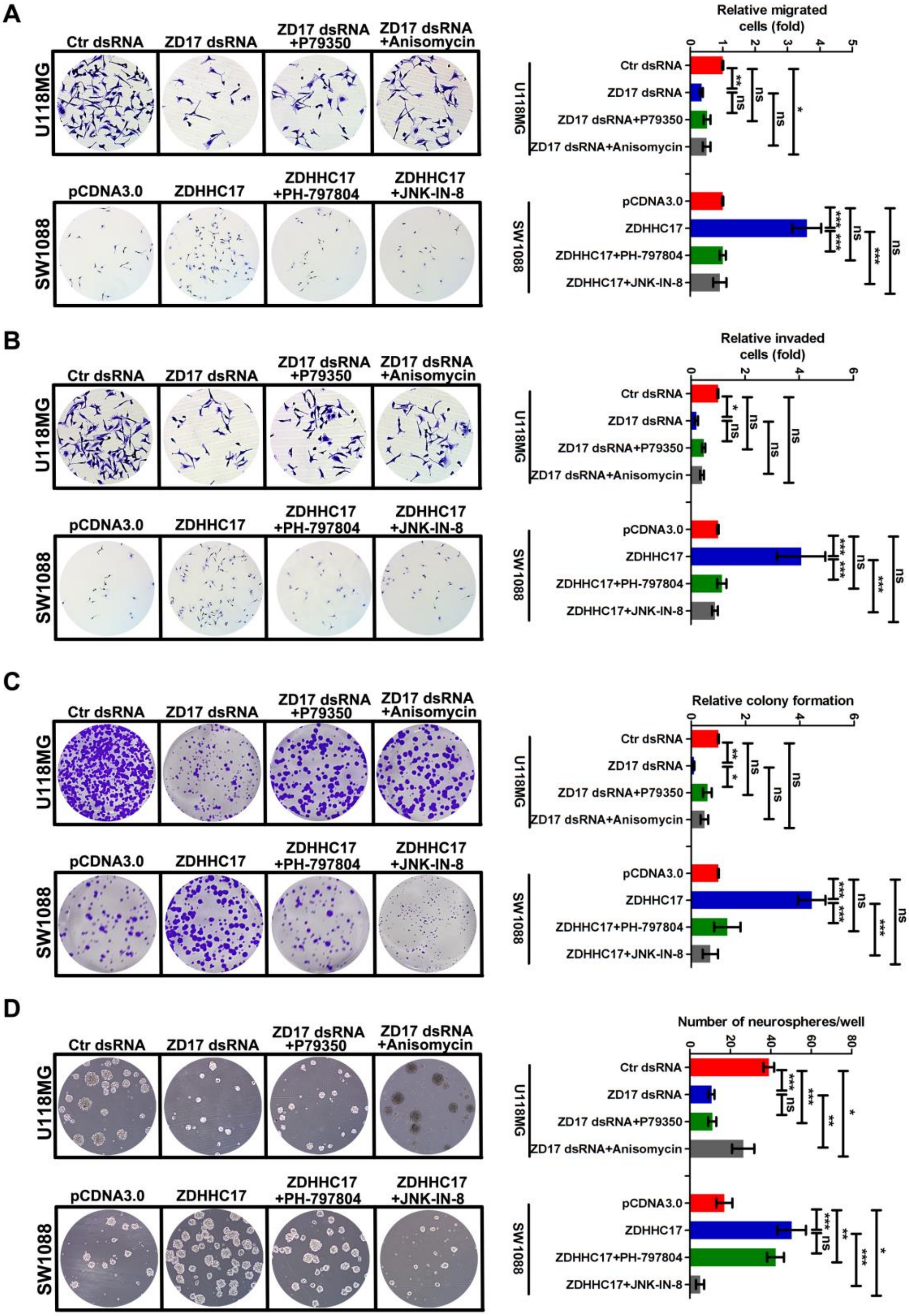

Supplementary Figure 11. JNK/p38 Activation is a Key Effector in ZDHHC17-Medited Glioblastoma Multiforme Malignant Progression

(A –D) Transwell analysis of migratory (A) and invasive (B) cells, or clonogenic survival of (C) or

self-renewal of glioma stem cells, as determined by number of neurospheres formed, from (D) U118MG or SW1088 cells transfected with indicated dsRNA, plasmid, or further treated with JNK agonist, anisomycin (10 μM); p38 agonist, P79350 (5 μM); *p*-JNK inhibitor, JNK-IN-8 (20 μM); or *p*-p38 inhibitor, PH-797804 (2.5 μM). Data represent the means ± SD from three separate experiments (*ns*, not significant; *$p < 0.05$; **$p < 0.01$; ***$p < 0.001$, unpaired *t* test).

Supplementary Figure 12

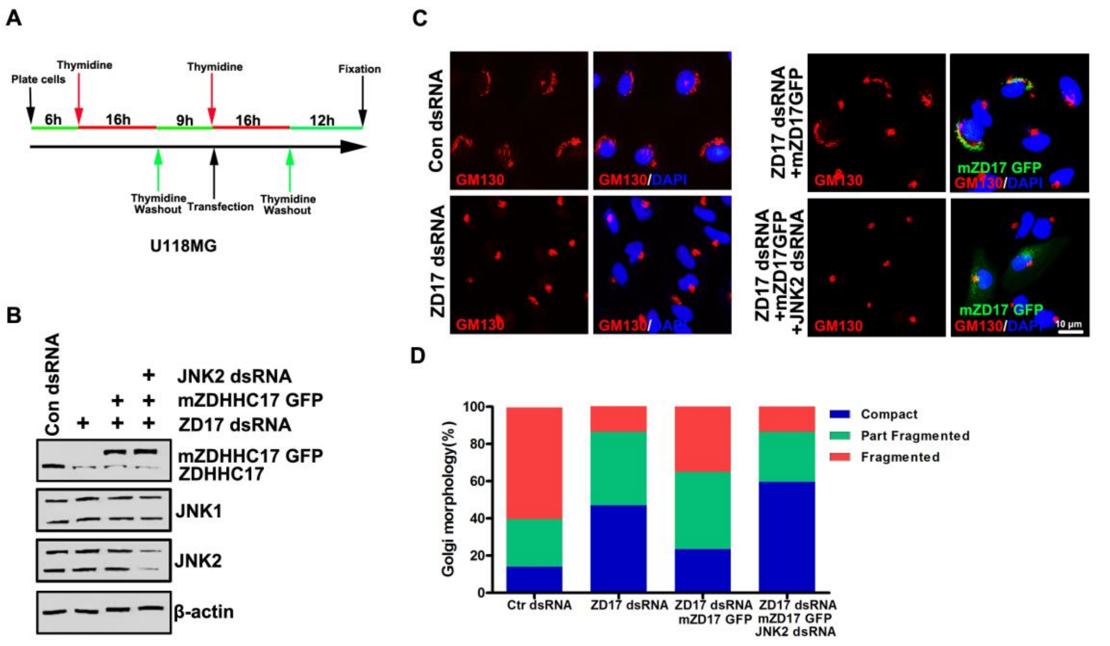

Supplementary Figure 12. ZDHHC17-JNK2 Signaling Module Controls Golgi Complex Fragmentation and Cell Cycle Progression

(A) Experimental scheme and pattern diagrams. U118MG cells were cultured on coverslips and synchronized in S phase with a double-thymidine block. At 1 h after the second-thymidine washout, the cells were transfected with indicated dsRNA, or plasmid, and fixed at the mitotic peak.

(B) Western blot of JNK1 and JNK2 in U118 MG cells transfected with control or ZDHHC17 dsRNA, or further transfected with mouse GFP-tagged ZDHHC17 plasmid or JNK2 dsRNA. β-actin was used as a loading control.

(C, D) Fixed cells were labeled with the anti-GM130 antibody to analyze Golgi membrane organization. Representative images of broken Golgi in G2 and of dense Golgi in U118 MG cells transfected with control or ZDHHC17 dsRNA, or further transfected with mouse GFP-tagged ZDHHC17 plasmid or JNK2 dsRNA . Scale bars, 10 μm. Quantification of cells treated as in C, including compact, fragmented, and partially fragmented Golgi. Results were calculated as the means ± SD from three independent experiments, with 200 cells counted per condition.

Supplementary Figure 13

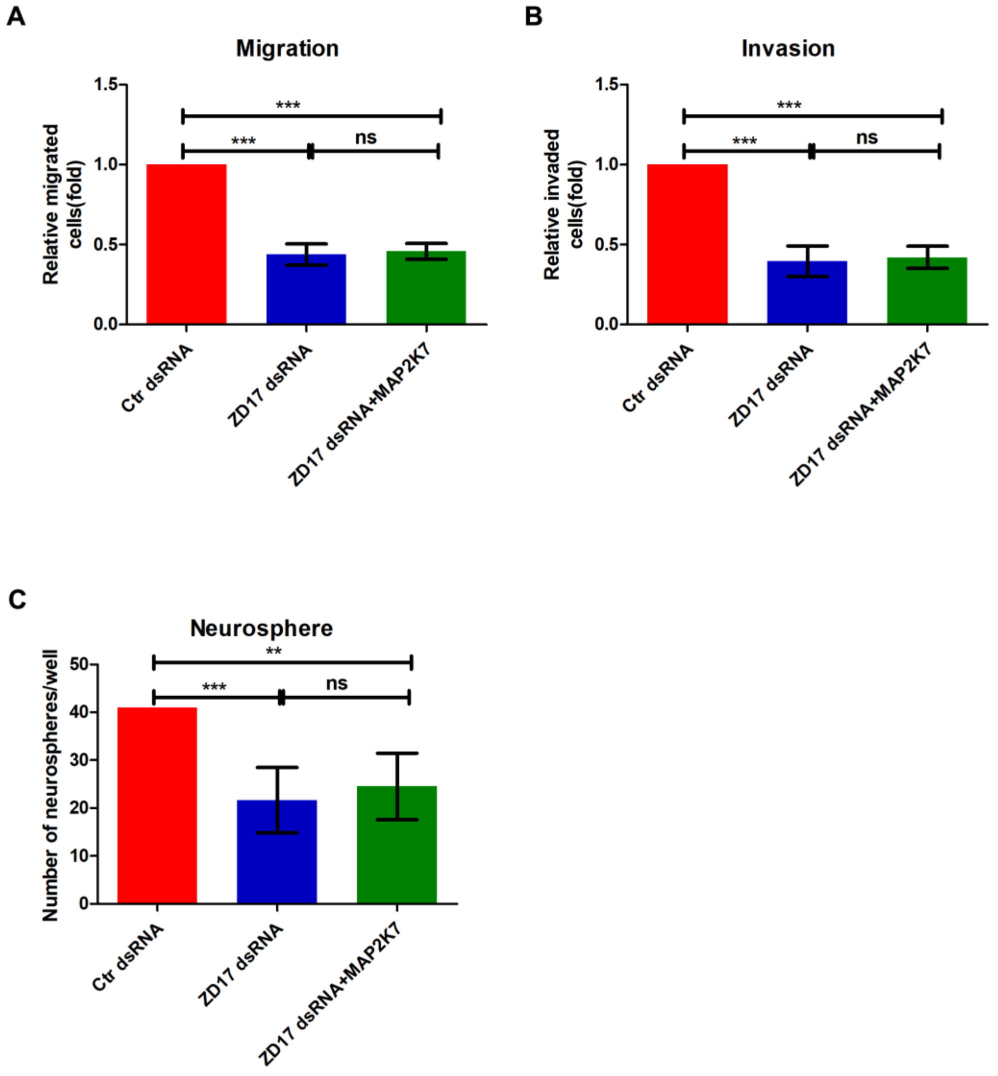

Supplementary Figure 13. Over-expression of MAP2K7 is not Sufficient to Rescue the Invasion, Migration and Self-renewal Phenotypes of GSCs after ZDHHC17 Knockdown

(A, B) Transwell analysis of migratory (A) and invasive (B) U118MG cells transfected with control, ZDHHC17 dsRNA, or further transfected with MAP2K7 plasmid. Data represent the means ± SD from three separate experiments (*ns*, not significant; $**p < 0.01$; $***p < 0.001$, unpaired *t* test).

(C) Neurosphere formation capacity of GSCs originating from U118MG cells transfected with control, ZDHHC17 dsRNA, or further transfected with MAP2K7 plasmid. Data represent the means ± SD from three separate experiments (*ns*, not significant; $***p < 0.001$, unpaired *t* test).
